# Osmotic and pH Stress‐Responsive Two‐Component System, OmpR/EnvZ, Modulates Type III Secretion, Biofilm Formation, Swimming Motility and Virulence in *Acidovorax citrulli* xjL12

**DOI:** 10.1111/mpp.70107

**Published:** 2025-06-16

**Authors:** Yuanjie Wang, Chenchao Sun, Ling Cai, Shitong Wu, Wenxin Chen, Yanli Tian, Baishi Hu, Ron Walcott

**Affiliations:** ^1^ College of Plant Protection and key Laboratory of Integrated Management of Crop Diseases and Pests Nanjing Agricultural University Nanjing China; ^2^ Key Laboratory of Plant Quarantine Pests Monitoring and Control Ministry of Agriculture and Rural Affairs Nanjing China; ^3^ Key Laboratory of Integrated Pest Management on Crops in Northwestern Oasis, Ministry of Agriculture and Rural Affairs, Xinjiang Key Laboratory of Agricultural bio‐Safety, Institute of Plant Protection Xinjiang Academy of Agricultural Sciences Urumqi Xinjiang China; ^4^ Department of Plant Pathology University of Georgia Athens Georgia USA

**Keywords:** *Acidovorax citrulli*, OmpR/EnvZ, stress response, type III secretion system, virulence regulation

## Abstract

*Acidovorax citrulli* , the causal pathogen of bacterial fruit blotch of cucurbits, relies on a functional type III secretion system (T3SS) for pathogenicity. Two‐component systems (TCSs) are primary signal transduction mechanisms for bacteria to detect and adapt to various environmental conditions. However, the role of TCS on regulating T3SS and other virulence factors in response to environmental stimuli is still poorly understood in *A. citrulli* . Here, we report the identification of a conserved TCS, OmpR/EnvZ, involved in hypersensitive response (HR) induction in *Nicotiana benthamiana* by screening a transposon‐insertion library in the group II strain xjL12 of *A. citrulli* . Transcription analysis confirmed that OmpR_Ac_/EnvZ_Ac_ was upregulated in response to elevated osmotic pressure, low and high pH conditions, and host environment. Deletions of *envZ*_*Ac*_, *ompR*_*Ac*_, or both *envZ*_*Ac*_ and *ompR*_*Ac*_ in *A. citrulli* attenuated virulence to melon seedlings and mature leaf tissues, and delayed HR in *N. benthamiana*. OmpR_Ac_ was activated by EnvZ_Ac_ and directly bound to the promoter region of *hrpG*, a major regulator of T3SS. This binding activated *hrpG* transcription and promoted T3SS assembly in T3SS‐inducing medium, XVM2. Additionally, the OmpR_Ac_/EnvZ_Ac_ mutants of *A. citrulli* displayed reduced swimming motility due to impaired flagella formation, but also had enhanced biofilm formation and exopolysaccharide production. OmpR_Ac_/EnvZ_Ac_ regulation of these virulence factors in *A. citrulli* depended on its own conserved phosphorylation sites. This work illuminates a signalling pathway for regulating the T3SS and provides insights into the OmpR/EnvZ‐mediated virulence regulatory network in *A. citrulli* .

## Graphical abstract

*Acidovorax citrulli* OmpR/EnvZ responds to extracellular pH and osmolarity, controls the T3SS by binding to the *hrpG* promoter, and is involved in regulating flagellar biogenesis, biofilm, and exopolysaccharide production.

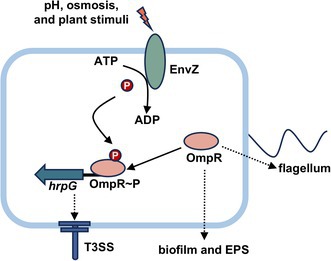

## 1 Introduction

Bacterial fruit blotch (BFB) caused by *Acidovorax citrulli* poses a serious threat to cucurbit (e.g., watermelon, melon) production worldwide. This pathogen is seed‐borne and seed‐transmitted, and contaminated seeds are often the primary source of inoculum for BFB epidemics (Burdman and Walcott 2012). *A. citrulli* employs diverse virulence factors, including secretion systems, polar flagellum, type IV pili, and biofilm formation, to successfully infect plant hosts (Bahar et al. 2009, 2011; Burdman and Walcott 2012; Kan et al. 2023; Tian et al. 2015). Like other gram‐negative, phytopathogenic bacteria, the type III secretion system (T3SS) in *A. citrulli* is required for infecting host plants and eliciting a hypersensitive response (HR) on non‐host plants (Burdman and Walcott 2012). *A. citrulli* directly injects a variety of T3SS effectors (T3Es) into plant cells via the T3SS to subvert the plant immune response and other cellular bioprocesses (Jiang et al. 2022; Jiménez‐Guerrero et al. 2020, 2024; Yang et al. 2023; Zhang et al. 2020). The hypersensitive response and pathogenicity (*hrp*) genes encoding the components of *A. citrulli* T3SS are controlled by the HrpG‐HrpX pathway, by which the AraC‐type transcriptional activator, HrpX, activated by the OmpR‐type regulator, HrpG, directly regulates the *hrp* cluster and some T3Es (Zhang et al. 2018).

T3SS gene expression is depressed in nutrient‐rich medium and significantly upregulated in plant tissues and in nutrient‐poor medium such as XVM2 (Huynh et al. 1989; Schulte and Bonas 1992). To be successful, pathogenic bacteria need to stringently regulate the expression of T3SS genes in response to different environmental conditions. In *A. citrulli* , histidine kinase BarA_Ac_, the homologue of GacS in *Pseudomonas syringae* , was identified as a T3SS regulator located upstream of the T3SS regulatory cascade (Qiao et al. 2022). BarA_Ac_ decreases the translation level of HrpG in rich medium through an unidentified mechanism (Qiao et al. 2022). Nevertheless, the precise regulation of T3SS in *A. citrulli* remains largely obscure.

Two‐component regulatory systems (TCSs) are common signal transduction mechanisms by which bacteria sense surrounding stimuli and acclimate to their natural habitats (Krell et al. 2010). A canonical TCS is composed of a histidine kinase (HK), containing a conserved kinase core, and a cognate response regulator (RR), containing a conserved N‐terminal receiver domain (REC) and a variable C‐terminal effector domain (Papon and Stock 2019; Stock et al. 2000). The membrane‐embedded HKs often detect extracellular signals through their periplasmic sensor and undergo autophosphorylation of a conserved histidine residue. The phosphoryl group is then transferred to a conserved aspartate residue within the REC domain of the cognate RR, finally activating the RR to trigger cellular adaptive responses. This response often alters transcriptional patterns of target genes (Gao et al. 2019; Stock et al. 2000).

One of the most extensively studied TCSs is OmpR/EnvZ, which is ubiquitous in gram‐negative bacteria and is renowned for functioning as an osmosensor in various bacteria (Ames et al. 1999; Cai and Inouye 2002). Under increasing osmolality, the response regulator, OmpR, is phosphorylated by its cognate sensor kinase, EnvZ, and activates or represses transcription of porin genes (Foo et al. 2015). Beyond osmoregulation, this TCS also plays a role in regulating the outer membrane porins or other cellular components in response to a spectrum of external stresses, including pH, temperature, oxidative stress and antibiotic exposure (Forst and Inouye 1988; Ji et al. 2024; Nieckarz et al. 2016; Xiao et al. 2022; Wang et al. 2023). Additionally, an array of studies demonstrated that OmpR/EnvZ is involved in controlling virulence factors, such as motility, biofilm formation, exopolysaccharides (EPS) and secretion systems. For example, in the plant pathogen *Erwinia amylovora* , OmpR/EnvZ negatively regulates the T3SS and amylovoran production, and positively regulates motility (Li et al. 2014). Shao et al. (2021) found that deletion of *ompR/envZ* resulted in higher levels EPS production and reduced motility in *P. syringae* and proved that OmpR/EnvZ is a repressor of the *P. syringae* T3SS in rich medium by indirectly regulating the T3SS regulator HrpR. Furthermore, OmpR/EnvZ and its homologues have a role in maintaining cellular iron homeostasis by directly regulating porins or siderophores (Gerken et al. 2020; Song et al. 2024). Although OmpR/EnvZ has been proven as a pleiotropic regulator, its role in the intricate regulatory network of virulence in *A. citrulli* has yet to be characterised.

Here, we identify a histidine kinase mutant obtained by random Tn*5* transposon insertion mutagenesis in the group II strain xjL12 displaying a compromised HR‐induction phenotype in *Nicotiana benthamiana* leaves. This kinase shares high sequence identity to a known EnvZ and constituting a TCS (named as OmpR_Ac_/EnvZ_Ac_) with its cognate response regulator. Phenotypic profiling showed that OmpR_Ac_/EnvZ_Ac_ responded to extracellular pH, osmolality, and host environment, contributed to *A. citrulli* virulence, positively regulated T3SS gene expression and swimming motility, while negatively controlling biofilm formation. Additionally, phosphorylated OmpR_Ac_ directly bound to the *hrpG* promoter to activate the HrpG regulon. Overall, our findings suggested that OmpR_Ac_/EnvZ_Ac_ is a global regulator in *A. citrulli* and provided new insights into the mechanism underlying the regulation of *A. citrulli* T3SS coordinated by a variety of environmental signals and host factors.

## 2 Results

### 2.1 Aave_1583 and Aave_1584 Constitute a Putative TCS of OmpR/EnvZ Family

**FIGURE 1:**
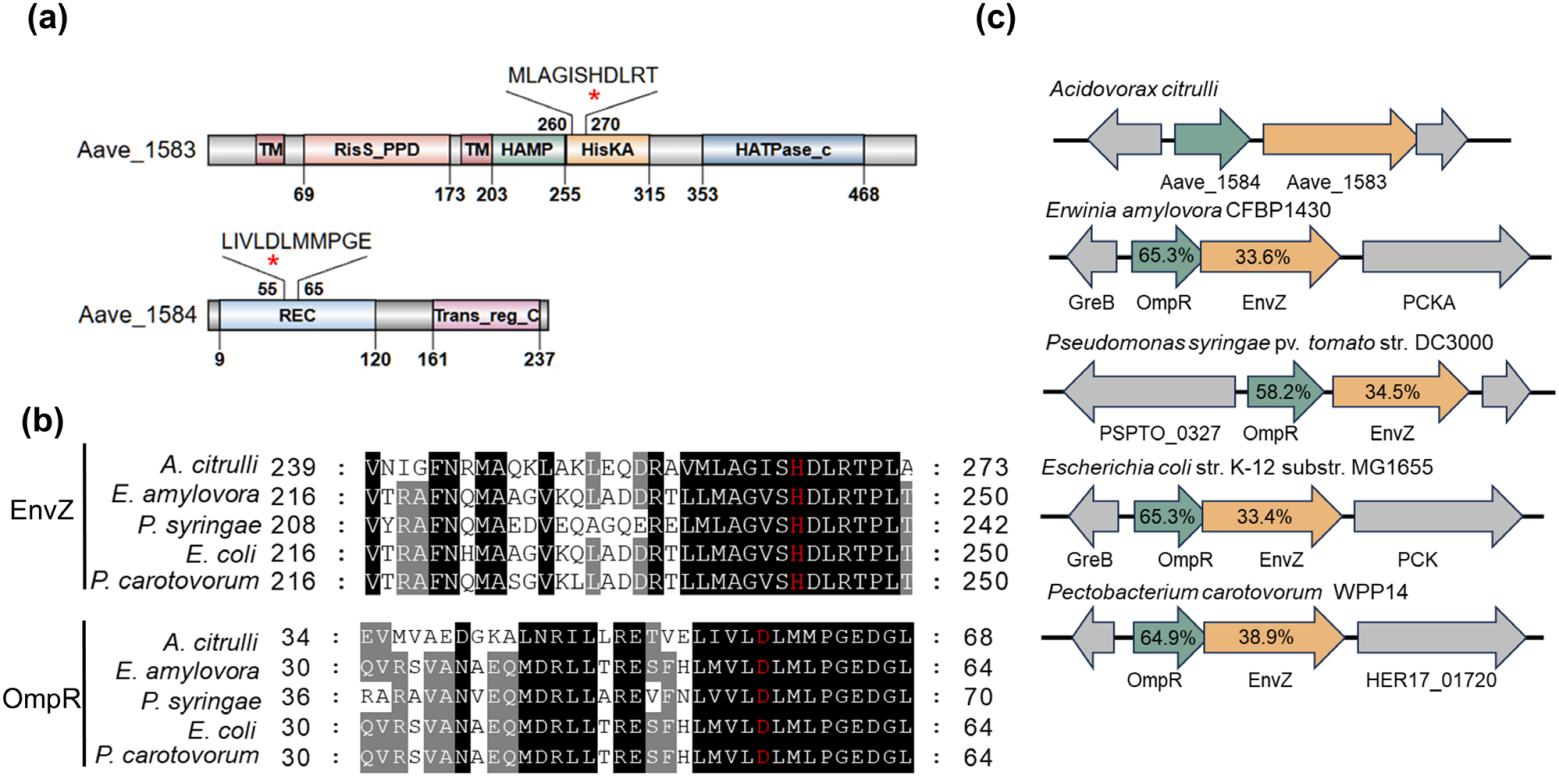
Genomic loci, sequence alignment and predicted secondary structures of Aave_1583 and Aave_1584. (a) Schematic representation of protein domains predicted by SMART. The predicted phosphorylation site indicated by a red star. TM, transmembrane region. HAMP, histidine kinases, adenylyl cyclases, methyl binding proteins, phosphatases domain. HisKA, His Kinase A (phosphoacceptor) domain. HATPase_C, histidine kinase‐like ATPases. REC, receiver domain. Trans_reg_C, C terminal of transcriptional regulatory protein. (b) Prediction of Aave_1583 and Aave_1584 phosphorylation sites in *Acidovorax citrulli* by aligning Aave_1583, Aave_1584 with their homologues. The analysis was performed by COBALT. Conserved and highly homologous residues are marked with black and grey, respectively. Residues in red indicate the putative phosphorylation site in EnvZ and OmpR. (c) Schematic representation of the genomic loci of *Aave_1584*/*Aave_1583* operon and its homologues in diverse genera. The percentages in arrows represent the amino acid identity of Aave_1584/Aave_1583 against homologues by BLASTP. Accession numbers (GenBank) of OmpR/EnvZ homologues: *A. citrulli* , WP_011794721/WP_011794720; *Erwinia amylovora* CFBP1430, CBA23707/CBA23705; *Pseudomonas syringae* pv. *tomato* DC3000, AAO53873/AAO53874; *Escherichia coli* K‐12 MG1655, NP_417864/NP_417863; *Pectobacterium carotovorum* WPP14, WP_005969424/WP_029368754.

A mutant with reduced ability to elicit HR in *N. benthamiana* was obtained through screening a Tn*5* transposon‐insertion *A. citrulli* xjL12 mutant library (Figure S1). The disrupted gene exhibits 100% sequence identity with *Aave_1583* in AAC00‐1, the type strain of group II, by sequencing and alignment analysis (Figure S2). *Aave_1583* encodes a sensor histidine kinase containing two transmembrane domains; a sensor domain, RisS_PPD, for signal recognition; a HAMP domain functioning as a signalling module in diverse signalling proteins; a HisKA domain containing a conserved His residue for autophosphorylation; and a HATPase_C domain, for catalysing ATP. Its upstream gene, *Aave_1584*, encodes a potential response regulator, consists of an N‐terminal REC domain for accepting phosphoryl group and a C‐terminal Trans_reg_C domain for DNA binding (Figure 1a). Conserved phosphorylation sites His266 and Asp59 were predicted within the HisKA domain of Aave_1583 and REC domain of Aave_1584, respectively (Figure 1a,b). Reverse transcription (RT)‐PCR analysis showed that a fragment of DNA spanning the intergenic region was amplified from cDNA (Figure S3a). This indicated that both genes are transcribed together as part of an operon. These findings suggested that Aave_1583 and Aave_1584 constituted a TCS in *A. citrulli* . Comparison of amino acid sequences revealed that Aave_1583 and Aave_1584 shared similarity to the putative OmpR/EnvZ pairs from *E. amylovora* , *P. syringae* , *Escherichia coli* and *Pectobacterium carotovorum* (Figure 1c). Accordingly, these genes were designated *ompR*_*Ac*_/*envZ*_*Ac*_. An unrooted phylogenetic tree was constructed for OmpR_Ac_/EnvZ_Ac_ and other OmpR/EnvZ homologues from 13 pathogenic bacterial species to further investigate genetic relationship. Putative OmpR/EnvZs from *Acidovorax* spp. show a close relationship with those from *Ralstonia solanacearum* , but a relatively distant evolutionary relationship with the well‐studied OmpR/EnvZs from *E. coli* and *Vibrio cholerae* (Figure S3b,c). Moreover, only *Acidovorax* spp. and *R. solanacearum* contain the sensor domain, RisS_PPD (PF16524), potentially allowing the sensing the pH of the environs (Figure S3c).

### 2.2 OmpR_Ac_ /EnvZ_Ac_ Responds to pH, High Osmotic Stress and Host Environment

**FIGURE 2:**
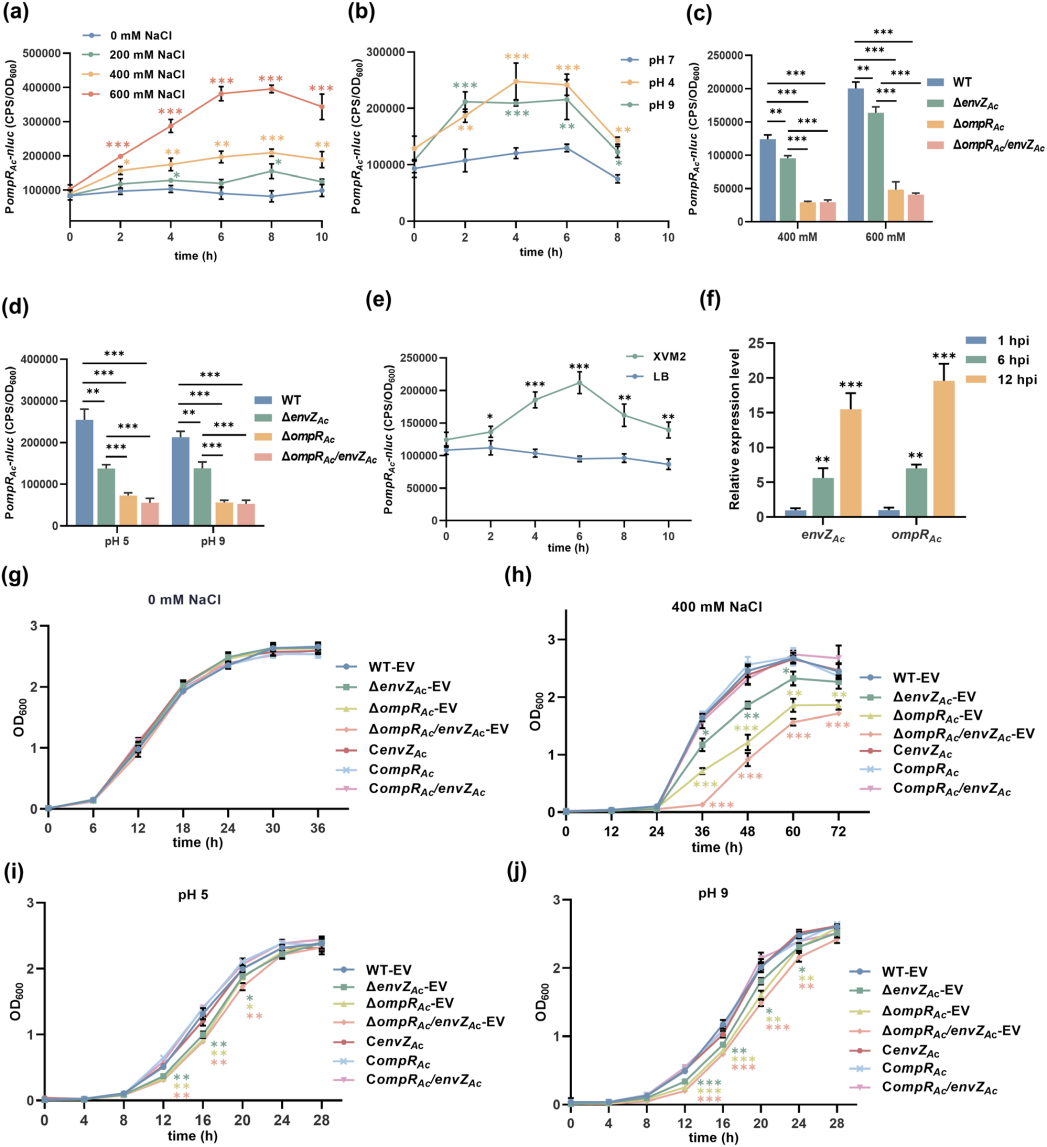
OmpR_Ac_/EnvZ_Ac_ was involved in the response of *Acidovorax citrulli* xjL12 to extracellular osmotic, acidic/alkaline stress and plant environment. (a, b) The transcriptional activity of *ompR*_*Ac*_ */envZ*_*Ac*_ operon in *A. citrulli* under osmotic pressure (a) and acidic/alkaline stress (b) was determined by Nanoluc (*nluc*) luciferase reporter system. The transcriptional level of *nluc* was controlled by the *ompR*_*Ac*_ promoter (P*ompR*_*Ac*_) in reporter vector pBBR‐P*ompR*_*Ac*_‐*nluc*. (c, d) The regulation of OmpR_Ac_/EnvZ_Ac_ with its own promoter activity under osmotic pressure (c) and pH stress (d). Promoter activity of transcriptional fusion, P*ompR*_*Ac*_‐*nluc* was detected at 4 h (c) and 2 h (d) after the strains were subjected to the indicated stressors. (e) The transcriptional activity of *ompR*_*Ac*_ */envZ*_*Ac*_ operon in XVM2 and Luria Bertani (LB) medium. (f) The relative expression level of *ompR*_*Ac*_ and *envZ*_*Ac*_ in melon cotyledons inoculated with xjL12 at 1, 6 and 12 h post‐inoculation (hpi). (g, h) Effect of OmpR_Ac_/EnvZ_Ac_ on xjL12 growth under 0 mM NaCl (g) and 400 mM NaCl (h). (i, j). Effect of OmpR_Ac_/EnvZ_Ac_ on xjL12 growth under pH 5 (i) and pH 9 (j). In (a–e), the reporter vector pBBR‐P*ompR*_*Ac*_‐*nluc* was introduced into wild‐type (WT) strain xjL12 and mutants, and the transcriptional activity *ompR*_*Ac*_ */envZ*_*Ac*_ operon was indirectly represented by fluorescence intensity, which was measured as counts per second (CPS) of light production. In (g–j), the population growth of *A. citrulli* strains in LB with indicated pH and NaCl concentration inferred by measured at an optical density (OD) of 600 nm with spectrophotometer. Error bars represent standard deviations of the means from three independent experiments. Asterisks indicate significant differences compared with the control (Student's *t* test. *p < 0.05, **p < 0.01, ***p < 0.001). In (a), (b) and (g–j), data points with not significant differences are unmarked. Different coloured asterisks correspond to different coloured lines. WT‐EV: Wild‐type strain xjL12 harbouring the empty vector pBBR1MCS‐5 (EV); Δ*envZ*_*Ac*_‐EV, Δ *ompR*_*Ac*_‐EV and Δ *ompR*_*Ac*_ */envZ*_*Ac*_‐EV: *EnvZ*_*Ac*_, *ompR*_*Ac*_ single mutant and dual mutant strain harbouring the empty vector pBBR1MCS‐5, respectively; C*envZ*_*Ac*_, C*ompR*_*Ac*_, C*ompR*_*Ac*_ */envZ*_*Ac*_: Complemented strain of Δ*envZ*_*Ac*_, Δ*ompR*_*Ac*_ and Δ*ompR*_*Ac*_ */envZ*_*Ac*_, respectively.

The well‐characterised TCS OmpR/EnvZ is known for its regulatory function in the expression of several genes in response to environmental changes in osmolarity, pH, temperature and nutrition (Cai and Inouye 2002; Nieckarz et al. 2016; Wang, Kuo, et al. 2021). To determine whether OmpR_Ac_/EnvZ_Ac_ is involved in response to osmolarity and pH, a Nanoluciferase (*nluc*) reporter plasmid, pBBR‐P*ompR*_*Ac*_‐*nluc*, was constructed to monitor the *ompR*_*Ac*_ */envZ*_*Ac*_ operon expression dynamics under high osmolality and acidic or alkaline conditions. The transcriptional levels of *nluc* are controlled by the native promoter of *ompR*_*Ac*_ (P*ompR*_*Ac*_) in the reporter plasmid. The transcriptional activity of P*ompR*_*Ac*_ was gradually increased with the rise in osmotic pressure induced by NaCl (Figure 2a). The activities of P*ompR*_*Ac*_‐*nluc* in 400 mM and 600 mM NaCl increased 2.18‐fold and 4.23‐fold at 6 h, respectively, compared to that in 0 mM NaCl. Subjected to acidic (pH 5) and alkaline (pH 9) stress, the transcriptional activity of P*ompR*_*Ac*_ was upregulated approximately 2‐fold compared to the response in pH 7 conditions (Figure 2b). RT‐quantitative PCR (RT‐qPCR) analysis confirmed the increased expression of *ompR*_*Ac*_ */envZ*_*Ac*_ under these conditions (Figure S4a,b). These results indicated that the *ompR*_*Ac*_ */envZ*_*Ac*_ operon is intrinsically upregulated in response to acidic, alkaline and high osmolality stress. In addition, under acidic, alkaline and high osmolality stress, the activity of P*ompR*_*Ac*_‐*nluc* was significantly decreased in the Δ*envZ*_*Ac*_, Δ*ompR*_*Ac*_ and Δ*ompR*_*Ac*_ */envZ*_*Ac*_ mutants compared to the parental strain, with Δ*ompR*_*Ac*_ and Δ*ompR*_*Ac*_ */envZ*_*Ac*_ exhibiting a significantly more pronounced reduction than Δ*envZ*_*Ac*_ (Figure 2c,d). This implied the potential regulation mediated by OmpR_Ac_/EnvZ_Ac_ in its self‐transcription. However, electrophoretic mobility shift assay (EMSA) analysis showed no DNA gel shift of P*ompR*_*Ac*_ after incubation with purified OmpR_Ac_ or OmpR_Ac_ phosphorylated by the cytoplasmic domain of purified EnvZ_Ac_ (EnvZ_Ac_C), indicating no direct interaction between P*ompR*_*Ac*_ and OmpR_Ac_ (Figure S5a).

To investigate whether *ompR*_*Ac*_ */envZ*_*Ac*_ responds to plant‐derived signals, the promoter activity of *ompR*_*Ac*_ in XVM2 medium simulating the plant apoplastic microenvironment was initially evaluated. The activation level of *ompR*_*Ac*_ was significantly increased in XVM2, compared to rich nutrient Luria Bertani (LB) medium (Figures 2e and S4c). Aberrant pH variations and hyperosmotic stress in XVM2 also enhanced the activity of P*ompR*_*Ac*_ (Figure S6). More importantly, the transcriptional level of *ompR*_*Ac*_ */envZ*_*Ac*_ progressively increased during xjL12 infection (Figure 2f). These indicated that *ompR*_*Ac*_ */envZ*_*Ac*_ plays an important role in adapting to the host environment.

Subsequently, we investigated the effect of OmpR_Ac_/EnvZ_Ac_ on *A. citrulli* growth under varying conditions by measuring temporal population dynamics of Δ*envZ*_*Ac*_, Δ*ompR*_*Ac*_ and Δ*ompR*_*Ac*_ */envZ*_*Ac*_ mutants, their complemented and wild‐type (WT) strains. No significant difference was found between the mutant and WT strains in low osmolality or conventional LB medium (Figures 2g and S7a). However, the mutants exhibited a disadvantage in growth in hyperosmotic medium containing 400 mM NaCl compared to that of the WT. This was especially the case with the double mutant, Δ*ompR*_*Ac*_ */envZ*_*Ac*_ (Figure 2h). The population growth of the mutants was attenuated in the exponential growth phase compared to that of the WT at pH 5 and pH 9 (Figure 2i,j). Furthermore, the absence of *envZ*_*Ac*_, *ompR*_*Ac*_ singly, and the double mutant led to no significant effect on the sensitivity of *A. citrulli* to high temperature and H_2_O_2_ (Figure S7b,c). Therefore, we inferred that OmpR_Ac_/EnvZ_Ac_ is critical for *A. citrulli* adaptation to elevated osmotic stress, but is not required for adaptation to acidic, alkaline, high temperature, and oxidative stress.

### 2.3 OmpR_Ac_ /EnvZ_Ac_ Positively Regulates *A. citrulli* xjL12 Virulence

Given that T3SS is indispensable in *A. citrulli* virulence and pathogenesis (Burdman and Walcott 2012), mutants consisting of in‐frame deletions of *envZ*_*Ac*_ and *ompR*_*Ac*_ were subjected to assays with several inoculation methods to determine the contribution of OmpR_Ac_/EnvZ_Ac_ to virulence.

**FIGURE 3:**
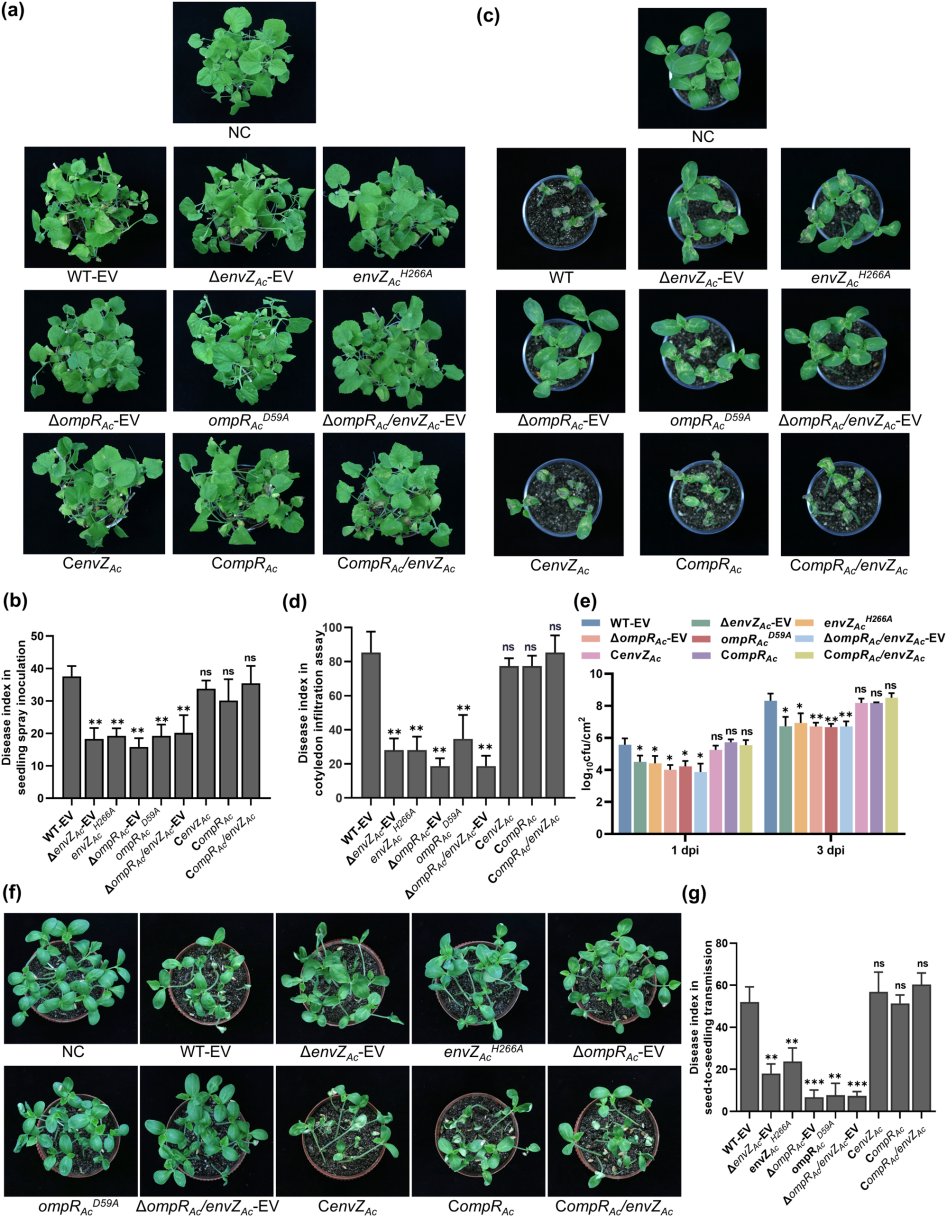
The effect of OmpR_Ac_/EnvZ_Ac_ on *Acidovorax citrulli* xjL12 virulence. (a) Approximately 3‐week‐old melon leaves were spray‐inoculated with *A. citrulli* strains. Cell suspensions (~10^8^ CFU/mL) were sprayed onto the leaves. The appearance of melon leaves was observed and photographed at 7 days post‐inoculation (dpi). (b) The disease index of spray‐inoculated leaves was investigated at 7 dpi. (c) Disease symptoms of 6‐day‐old melon seedlings inoculated with *A. citrulli*. Bacterial suspensions (~10^4^ CFU/mL) were syringe‐inoculated into cotyledons, and the appearance of seedlings was captured at 5 dpi. (d) The disease index of syringe‐inoculated melon cotyledons was investigated at 5 dpi. (e) The population of bacteria in melon cotyledon syringe‐inoculated with tested strains was quantified at 1 and 3 dpi. (f) Role of OmpR_Ac_/EnvZ_Ac_ in seed‐to‐seedling transmission of bacterial fruit blotch in melon. Twenty germinating melon seeds infiltrated with *A. citrulli* strains (~10^6^ CFU/mL) were planted per pot. The images were taken at 7 dpi. (g) The disease index of the seed‐to‐seedling transmission assay was investigated at 5 dpi. The error bars represent the standard deviation of the means from three independent experiments. Asterisks indicate significant differences compared with the wild‐type strain (Student's *t* test, *p < 0.05, **p < 0.01, ***p < 0.001; ns, no significance). NC, negative control with sterile water; WT‐EV: Wild‐type strain xjL12 harbouring the empty vector pBBR1MCS‐5 (EV); Δ*envZ*_*Ac*_‐EV, Δ *ompR*_*Ac*_‐EV and Δ *ompR*_*Ac*_ */envZ*_*Ac*_‐EV: *EnvZ*_*Ac*_, *ompR*_*Ac*_ single mutant and dual mutant strain harbouring the empty vector pBBR1MCS‐5, respectively; C*envZ*_*Ac*_, C*ompR*_*Ac*_ and C*ompR*_*Ac*_ */envZ*_*Ac*_: Complemented strain of Δ*envZ*_*Ac*_, Δ*ompR*_*Ac*_and Δ*ompR*_*Ac*_ */envZ*_*Ac*_, respectively; *envZ*_*Ac*_^H266A^: *EnvZ*_*Ac*_ point mutation with the His266 substituted by Ala; *ompR*_*Ac*_^D59A^: *OmpR*_*Ac*_ point mutation with the Asp59 substituted by Ala.

True leaves of melon plants spray‐inoculated with cell suspensions of Δ*envZ*_*Ac*_, Δ*ompR*_*A*c_ and Δ*ompR*_*Ac*_ */envZ*_*Ac*_ developed mild necrotic symptoms. The disease indices and bacterial populations of plants inoculated with mutants significantly decreased relative to those inoculated with the WT strain (Figures 3a,b and S8a). In addition, the disease indices of cotyledons of melon seedlings injected with three mutants were reduced to approximately less than half of that of the WT (Figure 3c,d). In agreement with the results of cotyledon injection assay, the populations of the mutant strains in melon cotyledons were significantly less than the WT strain at the first and third days post‐inoculation (Figure 3e), indicating that the absence of OmpR_Ac_/EnvZ_Ac_ impaired *A. citrulli* proliferation in melon cotyledon tissue. Genetic complementation of the mutants fully or partially restored virulence to WT levels (Figure 3a–e). These results suggested that the eliminating OmpR_Ac_/EnvZ_Ac_ substantially decreased *A. citrulli* virulence on melon plants.

To examine the effect of OmpR_Ac_/EnvZ_Ac_ on seed‐to‐seedling transmission, which is a primary characteristic of BFB as a seed‐transmitted disease, melon seeds were inoculated with WT, mutant, and complemented strains followed by planting. The seedlings that developed from melon seeds inoculated with *ompR*_*Ac*_ */envZ*_*Ac*_ mutants showed alleviated symptoms, while those from seeds inoculated with the WT strain developed necrosis or collapsed 1 week after planting (Figure 3f). The disease indices that resulted from inoculation with the *ompR*_*Ac*_ */envZ*_*Ac*_ mutants were significantly lower than that induced by the WT and complemented strains (Figure 3g). The *ompR*_*Ac*_ */envZ*_*Ac*_ mutants also exhibited significantly attenuated colonisation capacity within seeds compared to the WT (Figure S8b). These implied the important role of OmpR_Ac_/EnvZ_Ac_ in seed‐to‐seedling transmission of BFB. Furthermore, two point mutants in Δ*envZ*_*Ac*_ and Δ*ompR*_*Ac*_ backgrounds were constructed with the conserved phosphorylation residue, His266 of EnvZ_Ac_ and Asp59 of OmpR_Ac_ substituted by Ala (EnvZ_Ac_^H266A^ and OmpR_Ac_^D59A^) to generate constitutively dephosphorylated EnvZ_Ac_ and OmpR_Ac_. *A. citrulli* mutants *envZ*_*Ac*_^*H266A*^ and *ompR*_*Ac*_^*D59A*^ exhibited similar reduced virulence to Δ*envZ*_*Ac*_ and Δ*ompR*_*Ac*_ in all virulence assays, respectively (Figures 3 and S8), indicating His266 of EnvZ_Ac_ and Asp59 of OmpR_Ac_ are indeed functional active sites, and that the autophosphorylation of EnvZ_Ac_ and phosphorylation of OmpR_Ac_ are indispensable for *A. citrulli* virulence. Collectively, our data suggest that OmpR_Ac_/EnvZ_Ac_ positively regulates *A. citrulli* virulence, and this modulation depends on phosphorylation of OmpR_Ac_ and EnvZ_Ac_.

### 2.4 OmpR_Ac_ /EnvZ_Ac_ Regulates T3SS by Directly Mediating *hrpG* Transcription

**FIGURE 4:**
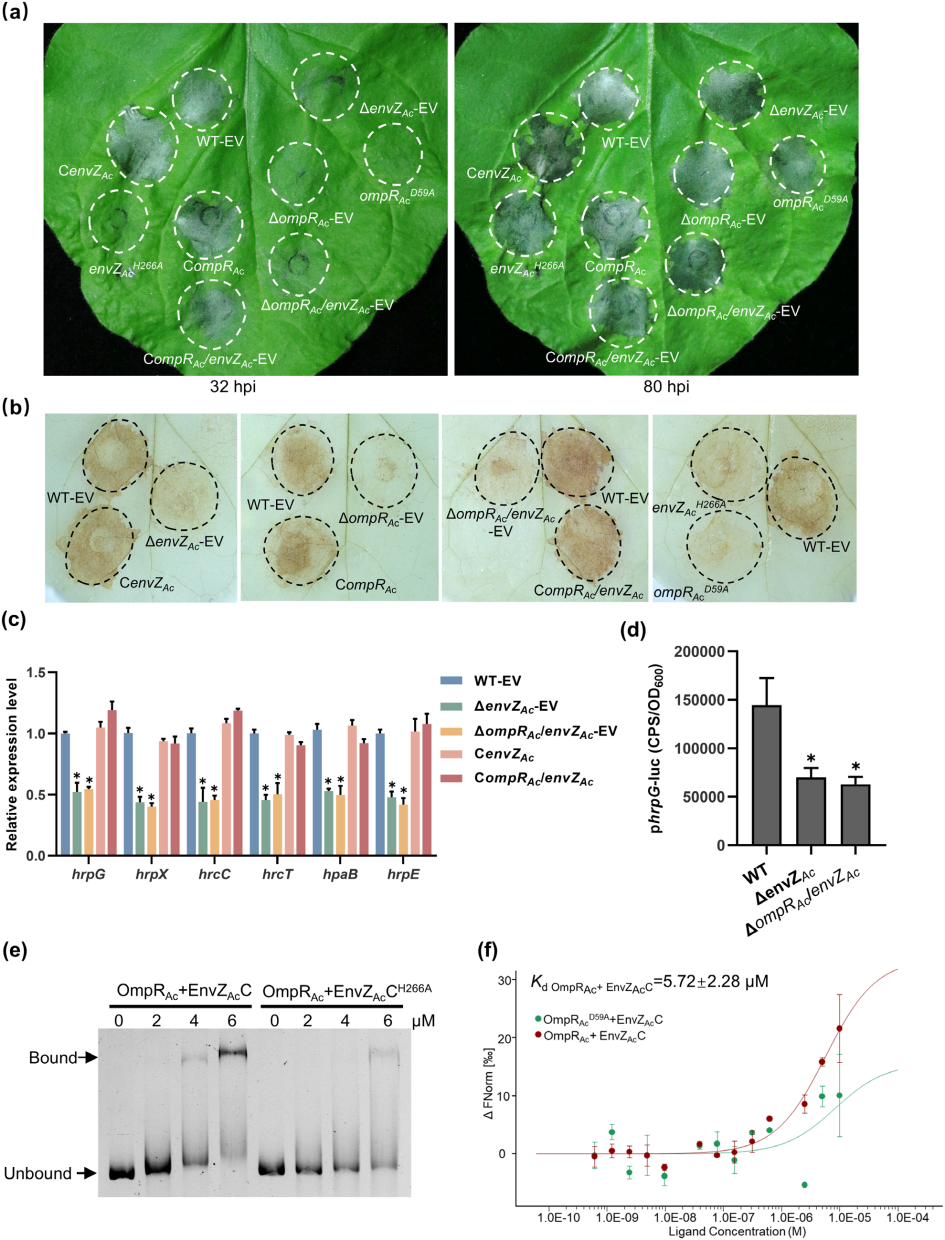
Dysfunctional OmpR_Ac_/EnvZ_Ac_ delayed hypersensitive response (HR) induction and OmpR_Ac_ directly bound to the promoter region of HrpG (P*hrpG*). (a) HR in non‐host, *Nicotiana benthamiana*. The cell suspensions at OD_600_ of 0.3 were infiltrated into the *N. benthamiana* leaves. Representative image was taken at 32 h and 80 h post‐infiltration (hpi). The inoculation areas were indicated with white dashed lines. (b) Reactive oxygen species (ROS) accumulation induced by mutations were assayed by 3,3′‐diaminobenzidine (DAB) staining. The *N. benthamiana* leaves injected with cell suspensions were sampled at 24 hpi, stained with DAB, and decolourised with 95% ethanol before taking pictures. The inoculation areas were indicated with black dashed lines. (c) The relative expression level of selected genes related to type III secretion system (T3SS) regulation and assembly. RNA was extracted from cells cultivated in T3SS‐inducing medium, XVM2. 16S ribosomal RNA gene was used as a reference gene. (d) Promoter activity of *hrpG* in the indicated strains. The recombinant plasmid containing transcriptional fusion P*hrpG*‐*nluc* was introduced into wild type (WT), Δ*envZ*_*Ac*_ and Δ*ompR*_*Ac*_ */envZ*_*Ac*_. Expression of P*hrpG*‐*nluc* from bacteria grown in XVM2 was measured as counts per second (CPS) of light production. (e) Electrophoretic mobility shift assay of OmpR_Ac_ with P*hrpG*. The purified OmpR_Ac_ protein was preincubated with purified EnvZ_Ac_C or EnvZ_Ac_C^H266A^ to activate regulator. Activated regulator (0–6 μM) and DNA fragment (50 ng) were incubated at room temperature for 20 min and then analysed by 6% polyacrylamide gel electrophoresis. The gels were stained by DNA dye and photographed by a gel imaging system. (f) Detection of OmpR_Ac_‐P*hrpG* binding with microscale thermophoresis (MST) analysis. The purified OmpR_Ac_ or OmpR_Ac_^D59A^ protein was preincubated with purified EnvZ_Ac_C to activate regulator. Response regulator (0.61 nM–10 μM) as ligand was incubated with Cy5‐labelled P*hrpG* in NT standard capillary in MST assays. *K*_d_, dissociation constant. WT‐EV: Wild‐type strain xjL12 harbouring the empty vector pBBR1MCS‐5 (EV); Δ*envZ*_*Ac*_‐EV, Δ*ompR*_*A*c_‐EV and Δ*ompR*_*A*c_ */envZ*_*Ac*_‐EV: *EnvZ*_*Ac*_, *ompR*_*A*c_ single mutant and dual mutant strain harbouring the empty vector pBBR1MCS‐5, respectively; C*envZ*_*Ac*_, C*ompR*_*A*c_ and C*ompR*_*A*c_ */envZ*_*Ac*_: Complemented strain of Δ*envZ*_*Ac*_, Δ*ompR*_*A*c_ and Δ*ompR*_*A*c_ */envZ*_*Ac*_, respectively; *envZ*_*Ac*_^H266A^: *EnvZ*_*Ac*_ point mutation with the His266 substituted by Ala; *ompR*_*A*c_^D59A^: *OmpR*_*A*c_ point mutation with the Asp59 substituted by Ala. In (c) and (d), asterisks indicate significant differences compared with the wild‐type strain (Student's *t* test, *p < 0.05). MST analysis was performed two replicates and other experiments were performed three times with similar results. Error bars represent standard deviations of replicates.

T3SS is an indispensable apparatus for most gram‐negative phytopathogenic bacteria to induce a HR on non‐host plants (Mudgett 2005). HR is a typical plant immune reaction characterised by a reactive oxygen species (ROS) burst. To explore the role of OmpR_Ac_/EnvZ_Ac_ on the *A. citrulli* T3SS, OmpR_Ac_/EnvZ_Ac_ mutants and their derived point mutants were syringe‐infiltrated into *N. benthamiana* leaves, a non‐host plant for *A. citrulli* . WT and complemented strains elicited a distinct HR at 32 h post‐inoculation (hpi), which was not observed with the mutants (Figure 4a). At 80 hpi, the areas infiltrated with mutant strains displayed an HR phenotype similar to that of the WT (Figure 4a). Additionally, these mutants led to less ROS accumulation in inoculated leaves relative to the WT and complemented strains at 24 hpi (Figure 4b). These results indicated that OmpR_Ac_/EnvZ_Ac_ is necessary to elicit HR in non‐host plants and potentially for a functional T3SS.

The delayed HR phenotype described above led to the hypothesis that OmpR_Ac_/EnvZ_Ac_ positively regulates T3SS assembly during plant tissue infection. To test this hypothesis, we first detected the relative expression of T3SS‐related genes by RT‐qPCR. The T3SS apparatus genes including *hrpC*, *hrcT*, *hrpE* and *hrp* gene regulator, *hrpX*, and its upstream regulator, *hrpG*, were significantly downregulated in Δ*envZ*_*Ac*_ and Δ*ompR*_*Ac*_ */envZ*_*Ac*_ (Figure 4c). This indicated that these mutants were impaired in their T3SS. Correspondingly, the promoter activity of the *hrpG* promoter (P*hrpG*) in Δ*envZ*_*Ac*_ and Δ*ompR*_*Ac*_ */envZ*_*Ac*_ was decreased ~2‐fold relative to the WT strain (Figure 4d). Therefore, we reasoned that the transcriptional factor, OmpR_Ac_ directly regulates the expression of *hrpG*, thereby controlling T3SS. Different amounts of activated OmpR_Ac_ phosphorylated by its cognate kinase EnvZ_Ac_ and constitutively dephosphorylated OmpR_Ac_ (OmpR_Ac_^D59A^) were incubated with P*hrpG* followed analysis by EMSA. As expected, a detectable shift in the migration of P*hrpG* was observed after incubation with activated OmpR_Ac_, which was phosphorylated by purified EnvZ_Ac_C (Figure 4e). In contrast, P*hrpG* incubated with inactivate OmpR_Ac_, which reacted with EnvZ_Ac_C^H266A^, incapable of autophosphorylation, or with OmpR_Ac_^D59A^ showed faint shift bands (Figures 4e and S5b), indicating that activated OmpR_Ac_ had a higher affinity to P*hrpG* than OmpR_Ac_^D59A^. This result was further supported by microscale thermophoresis (MST) analysis. This analysis is used to quantify the binding affinity of molecular interactions by detecting alterations in thermophoresis induced by binding events (Asmari et al. 2018). OmpR_Ac_ phosphorylated by EnvZ_Ac_C directly bound to the Cy5‐labelled P‐*phrpG* (with *K*_d_ = 5.72 ± 2.28 μM), while OmpR_Ac_^D59A^ did not (Figure 4f). These results confirmed that OmpR_Ac_/EnvZ_Ac_ positively regulates the *A. citrulli* T3SS by direct binding to the promoter of *hrpG*.

### 2.5 OmpR_Ac_ /EnvZ_Ac_ Is Involved in Biofilm and Flagella Formation

**FIGURE 5:**
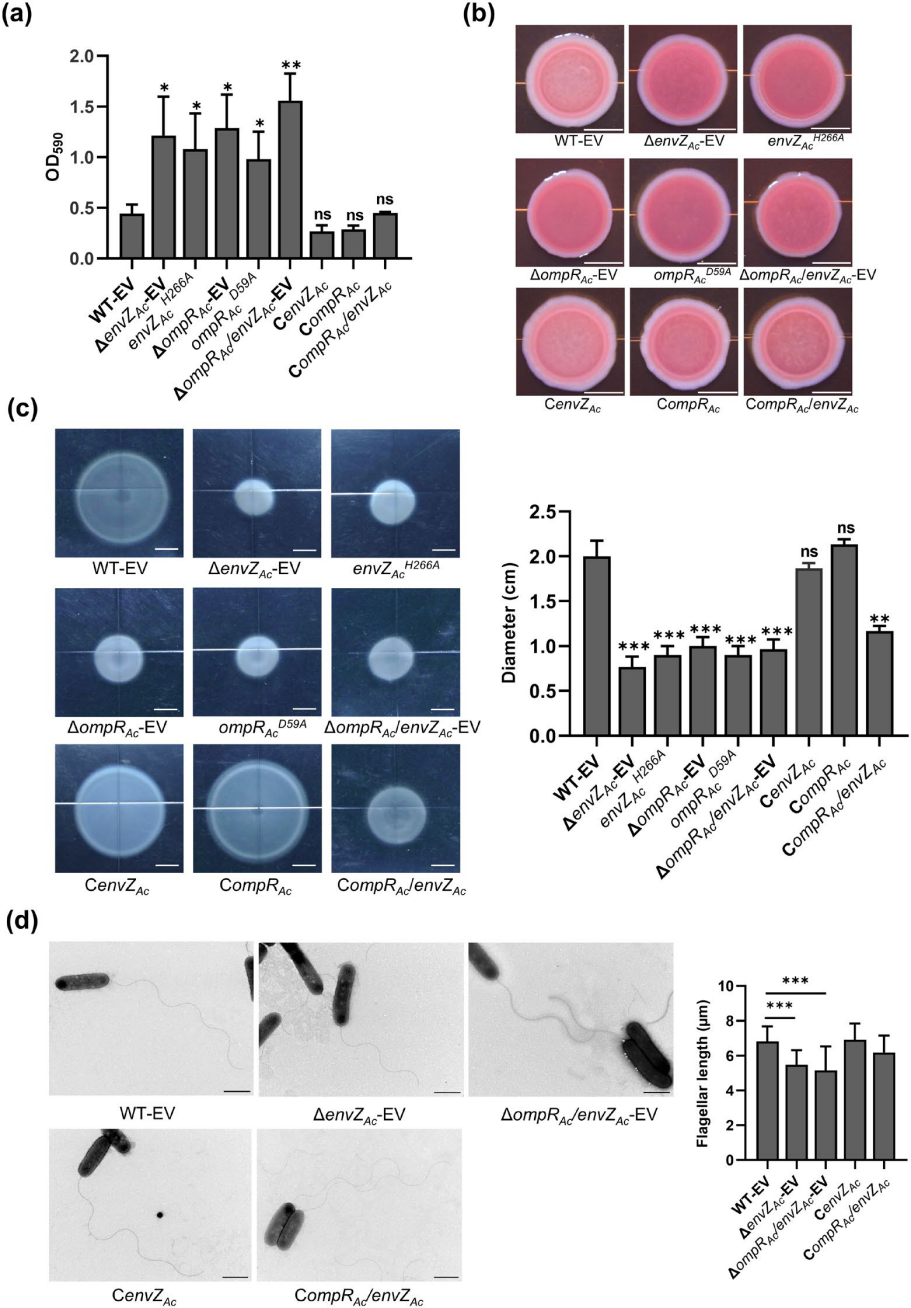
Effect of OmpR_Ac_/EnvZ_Ac_ on biofilm formation and swimming motility in *Acidovorax citulli* xjL12. (a) Quantification of biofilm. Biofilm was stained by crystal violet. Crystal violet biofilm was dissolved in 95% ethanol and quantified by spectrophotometer at OD_590_. (b) Morphotypes of colonies were investigated on Congo red agar plates. Bacteria with OD_600_ = 1.0 were spotted on plates containing Congo red. Representative images of Congo red‐stained colonies were captured after 4 days. Scale bar, 5 mm. (c) Swimming motility phenotypes. Cell suspension at OD_600_ of 0.3 was spotted on plates containing 0.3% agar and cultured for 36 h before taking pictures (left), and then the diameters of colonies were measured (right). Scale bar, 5 mm. (d) Bacterial flagella were observed by transmission electron microscopy. Scale bar, 1 μm. The flagellar lengths (*n* > 20) of each strain were measured by ImageJ software. The error bars represent the standard deviation of the means from three independent experiments. Asterisks indicate significant differences compared with the wild‐type strain (Student's *t* test, *p < 0.05, **p < 0.01, ***p < 0.001; ns, no significance). WT‐EV: Wild‐type strain xjL12 harbouring the empty vector pBBR1MCS‐5 (EV); Δ*envZ*_*Ac*_‐EV, Δ*ompR*_*Ac*_‐EV and Δ*ompR*_*Ac*_ /*envZ*_*Ac*_‐EV: *EnvZ*_*Ac*_, *ompR*_*Ac*_ single mutant and dual mutant strain harbouring the empty vector pBBR1MCS‐5, respectively; C*envZ*_*Ac*_, C*ompR*_*Ac*_ and C*ompR*_*Ac*_/*envZ*_*Ac*_: Complemented strain of Δ*envZ*_*Ac*_, Δ*ompR*_*Ac*_ and Δ*ompR*_*Ac*_ /*envZ*_*Ac*_, respectively; *envZ*_*Ac*_^H266A^: *EnvZ*_*Ac*_ point mutation with the His266 substituted by Ala; *ompR*_*Ac*_^D59A^: *OmpR*_*Ac*_ point mutation with the Asp59 substituted by Ala.

To further elucidate the roles of OmpR_Ac_/EnvZ_Ac_ in *A. citrulli* pathogenesis, we measured virulence‐associated biofilm and bacterial motility in OmpR_Ac_/EnvZ_Ac_ mutants. *A. citrulli* forms biofilms at the air–liquid interface, which can be stained by crystal violet and quantified. *ompR*_*Ac*_ */envZ*_*Ac*_ in‐frame mutants and point mutants increased biofilm formation > 2‐fold relative to WT and the complemented strains (Figures 5a and S9a). Although *A. citrulli* biofilm formation is limited in XVM2 minimal medium, the ability of *ompR*_*Ac*_ */envZ*_*Ac*_ mutants to form biofilm was enhanced significantly compared to the WT (Figure S9b). Biofilms are complex three‐dimensional multicellular aggregations usually encapsulated in a self‐produced matrix including EPS. EPS can be qualitatively assayed with Congo red dye in which Congo red preferentially binds to β‐(1,4)‐ and β‐(1,3)‐D‐glucans (Tosh 2013; Wood 1981; Wood et al. 1988). In this assay, colonies with overproduced EPS appear redder or more wrinkled, while EPS‐deficient colonies typically display reduced redness and/or wrinkling (Wang, Cady, et al. 2021). The centre and mid‐zone regions of *ompR*_*Ac*_ */envZ*_*Ac*_ mutant colonies cultured on Congo red agar plates were redder than those of the WT and complemented strains (Figures 5b and S10a), suggesting that the ability of mutants to produce EPS was enhanced. Swimming motility is a flagellum‐driven movement in liquid or low‐viscosity conditions. Deletions of *envZ*_*Ac*_, *ompR*_*A*c_, or both *ompR*_*Ac*_ and *envZ*_*Ac*_, and their derived point mutants displayed reduced swimming zones by approximately 2‐fold compared to WT strains (Figures 5c and S10b). Genetic complementation of mutants, except for Δ*ompR*_*Ac*_ */envZ*_*Ac*_, successfully restored the swimming motility to WT levels (Figure 5c). Transmission electron microscopy was performed to investigate flagella formation in mutants. The Δ*envZ*_*Ac*_ and Δ*ompR*_*Ac*_ */envZ*_*Ac*_ formed significantly shorter flagella than those observed in the WT strain, which confirmed the motility phenotype (Figure 5d). RT‐qPCR analysis revealed that the expression level of flagellum‐related genes markedly decreased in Δ*envZ*_*Ac*_ and Δ*ompR*_*Ac*_ */envZ*_*Ac*_ (Figure S4d). These results suggested that OmpR_Ac_/EnvZ_Ac_ negatively controls *A. citrulli* biofilm formation but positively controls swimming motility.

## 3 Discussion

**FIGURE 6:**
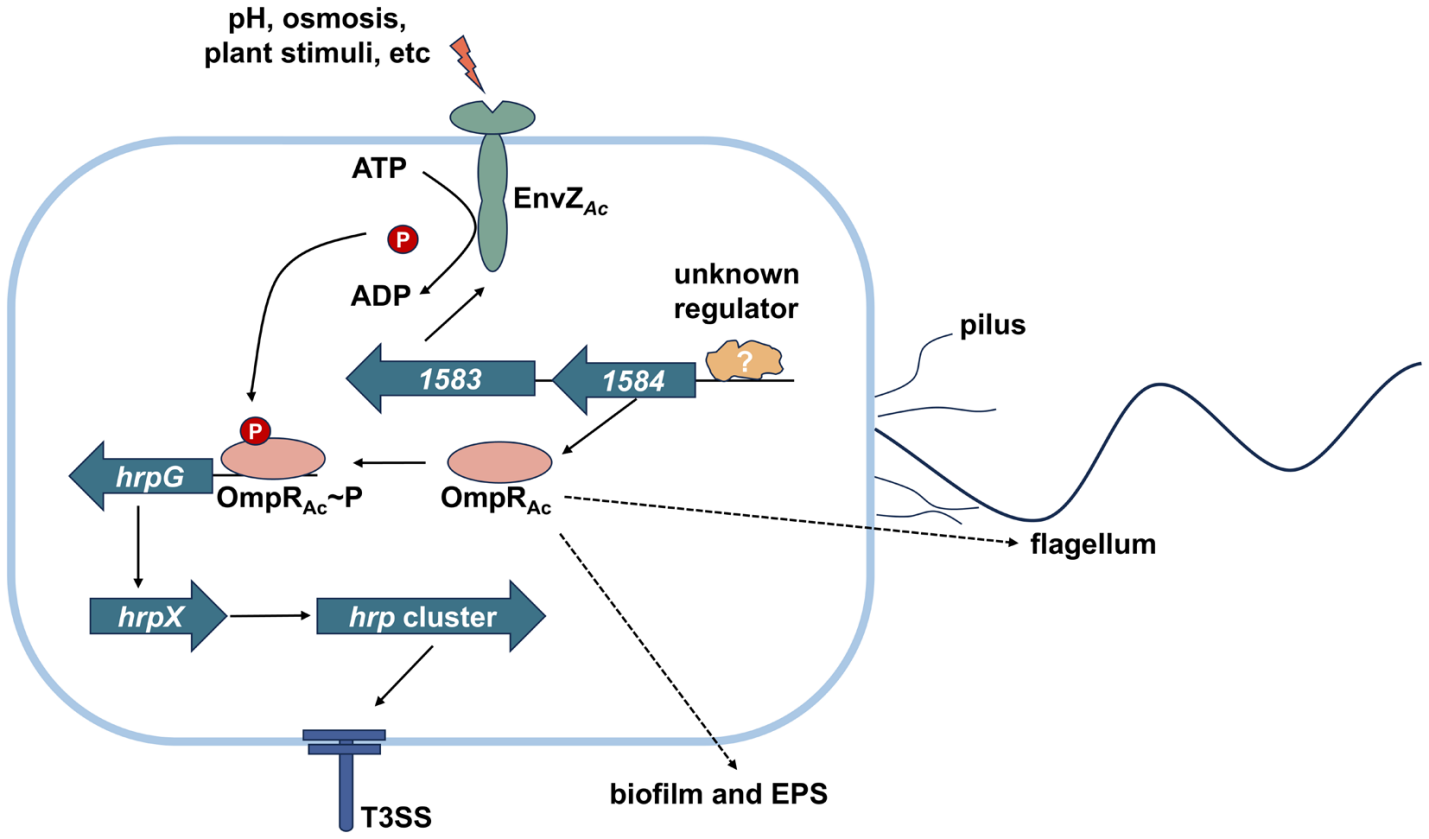
Schematic representation of the genetic regulation of OmpR_Ac_/EnvZ_Ac_ in *Acidovorax citrulli* . EnvZ_Ac_ undergoes autophosphorylation on its conserved histidine residue in response to external stimuli including extreme pH or elevated osmotic pressure. Subsequently, EnvZ_Ac_ transfers the phosphoryl group to response regulator OmpR_Ac_ (OmpR_Ac_ ~ *P*). Meanwhile, the transcription level of OmpR_Ac_ is upregulated, which is mediated by an unknown regulator. OmpR_Ac_ ~ *P* activates the transcription of type III secretion system (T3SS) regulator *hrpG* by specially binding to the promoter region of *hrpG*, thereby enhancing the expression of *hrpX* and *hrp* genes, and ultimately leading to the assembly of the T3SS. Additionally, OmpR_Ac_ or OmpR_Ac_ ~ *P* may also be involved in regulating flagella assembly, biofilm formation and exopolysaccharides (EPS) production by an uncharacterised mechanism.

Pathogens evolve membrane‐localised receptors with kinase activity to perceive dynamic environmental conditions, such as fluctuations in osmotic pressure or pH, and transmit these signals to cytoplasm through phosphotransfer reaction, thereby activating corresponding regulators to coordinate virulence factors and physiological metabolism. This results in adaption to the host environment and compromised host defences (Anderson 2023; Flint et al. 2016; Leonard et al. 2017). In this study, we discovered that the TCS OmpR_Ac_/EnvZ_*Ac*_ was crucial for pathogenicity and was activated under excessive osmotic pressure, acidic or alkaline conditions, and in planta. Phosphorylated OmpR_Ac_ directly bound to the promoter region of *hrpG*, the major regulator of T3SS in *A. citrulli* , promoting the *hrpG* transcription in T3SS‐inducing medium. Mutated OmpR_Ac_/EnvZ_*Ac*_ downregulated the expression of *hrp* genes and impaired the bacterium's ability to induce HR in non‐host plants. Deletion of *envZ*_*Ac*_, *ompR*_*Ac*_, or both *envZ*_*Ac*_ and *ompR*_*Ac*_ decreased flagella‐driven swimming motility, but enhanced biofilm formation, which may be attributed to increased EPS. Besides, the point mutants of predicted phosphorylation sites within EnvZ_*Ac*_ and OmpR_Ac_ displayed similar phenotypes as the in‐frame mutants of EnvZ_*Ac*_ and OmpR_Ac_. Based on the above results, we propose a model for OmpR_Ac_/EnvZ_Ac_‐mediated virulence regulatory network in *A. citrulli* . Osmotic stress, pH changes, and plant stimuli activate the transcription of *ompR*_*Ac*_ */envZ*_*Ac*_ operon, which facilitates the T3SS assembly through direct transcriptional activation of the *hrpG* promoter and is involved in regulating flagellar biogenesis, biofilm formation and EPS production in *A. citrulli* (Figure 6).

Many TCSs perform transcriptional autoregulation by binding their own promoters to generate required levels of active regulator, which are essential for executing the genetic regulation under certain conditions, and this regulation model allows bacteria to respond more rapidly and efficiently to environmental fluctuations (Groisman 2016). In this study, although the expression of the *ompR*_*Ac*_ */envZ*_*Ac*_ operon surged under high osmotic and acidic/alkaline pressure, neither OmpR_Ac_ nor activated OmpR_Ac_ showed high binding affinity with its promoter region, suggesting the potential presence of other regulators directly controlling the *ompR*_*Ac*_ */envZ*_*Ac*_ transcription. Intriguingly, the less pronounced reduction of P*ompR*_*Ac*_ activity in Δ*envZ*_*Ac*_ than Δ*ompR*_*Ac*_ and Δ*ompR*_*Ac*_/*envZ*_*A*c_ implied that, in addition to EnvZ_Ac_, other HKs could be involved in activating OmpR_Ac_ in response to external stressors. These findings suggested the intricacy of osmoregulation or pH regulation in *A. citrulli*. Moreover, it is noteworthy that the activity of RR with truncated HK in vitro may greatly differ from that of the full‐length HK under natural cellular conditions (Gao et al. 2019). Whether OmpR_Ac_ binds to its promoter in vivo remains to be investigated.

The selective pressures that bacteria encounter in different niches fuel the evolution of orthologous regulators to respond to new signals and to adjust the constituents of the regulon. Although OmpR/EnvZ is conserved in multiple bacteria, there are still differences in the environmental triggers of OmpR/EnvZ and in gene expression outputs (Kunkle et al. 2020; Quinn et al. 2014; Tipton and Rather 2017). For example, OmpR is identical for *Salmonella enterica* subsp. *enterica* serovar Typhimurium and *E. coli* , while OmpR levels are induced by acid conditions in *S. typhimurium*, but not in *E. coli* (Quinn et al. 2014). OmpR in *V. cholerae* is responsive to alkaline conditions, but not to osmolarity, the canonical stimulus for inducing OmpR (Kunkle et al. 2020). Instead, OmpR_Ac_ responds to a broad range of stimuli including osmolarity and pH. The specific RisS_PPD sensor domain in EnvZ_Ac_ raises a presumption that EnvZ_Ac_ may perceive some exclusive signals. In phytopathogenic bacteria, conserved OmpR also exhibits largely divergent regulons, as exemplified in regulating T3SS generally as a pathogenicity determinant for many pathogens. Previous studies established that OmpR in *P. syringae* and *E. amylovora* indirectly downregulated *hrp* genes in *hrp*‐inducing medium (Li et al. 2014; Shao et al. 2021). In contrast, our study demonstrates that OmpR_Ac_ positively controls the assembly of T3SS by directly regulating *hrp* gene regulator, HrpG in XVM2. We speculate that the differential regulatory pattern of T3SS mediated by OmpR could be due to the following: (i) the different habitat preferences, including host‐pathogenic interactions, which drive the evolution of OmpR; and/or (ii) the difference in *hrp* cluster, where the *hrp* clusters of *P. syringae* and *E. amylovora* are classified as group I, while that of *A. citrulli* is classified as group II (Alfano and Collmer 1996; Burdman and Walcott 2012).

Biofilms are multicellular communities encased in a self‐produced polymeric matrix. For pathogenic bacteria, the biofilm is conducive to coping with host defence responses, promoting colonisation. However, phenotypic profiling of mutants revealed two contradictory results, namely, enhanced biofilm formation and reduced virulence. In fact, similar phenotypes have been reported in several studies (McNally et al. 2012; Zeng et al. 2013; Zhu et al. 2011). The crystal violet staining described in this study allows for observing bacterial attachment and, presumably, biofilm formation to abiotic surfaces, but provides no evidence regarding the three‐dimensional structure and the biofilm formation process. The overproduction of EPS in the *ompR*_*Ac*_ */envZ*_*Ac*_ mutants, which paralleled observations reported in *P. syringae* and *E. amylovora* (Li et al. 2014; Shao et al. 2021), may give rise to the hyperattachment phenotype. An expanded model of biofilm formation involves three events: aggregation and attachment to biotic or abiotic surfaces, growth and accumulation, and finally, disaggregation and detachment from biofilm to the planktonic state (Sauer et al. 2022). The last event facilitates the dissemination of bacteria and subsequent colonisation of new sites (Purevdorj‐Gage et al. 2005). We put forward the hypothesis that although the dysfunctional OmpR_Ac_/EnvZ_Ac_ may reinforce attachment to the vascular tissue of melon, this hyperattachment could constrain dispersion from biofilm, thereby limiting transmission within the melon tissues. Additionally, the impaired flagella in OmpR_Ac_/EnvZ_Ac_ mutants may limit the in planta transmission, considering the role of polar flagellum in facilitating movement within the stem of melon seedlings (Bahar et al. 2011). Furthermore, the enhanced biofilm along with reduced swimming motility seems to demonstrate the preference for a sessile lifestyle in OmpR_Ac_/EnvZ_Ac_ mutants. Whether OmpR_Ac_/EnvZ_Ac_ controls the switch between a sessile and planktonic lifestyle merits further investigation.

In conclusion, we identified the TCS, OmpR/EnvZ involved in response to osmolarity, pH, and host environment as a T3SS regulator in *A. citrulli* . OmpR_Ac_/EnvZ_Ac_ directly controlled the T3SS genes expression level by OmpR_Ac_ binding to the promoter region of *hrpG* in a phosphorylation‐dependent manner. Further, OmpR_Ac_/EnvZ_Ac_ positively regulated the flagella assembly but negatively regulated the biofilm formation and EPS production. The molecular mechanism underlying OmpR_Ac_/EnvZ_Ac_‐mediated motility and biofilm needs to be deciphered in future. These findings expand our understanding of the regulatory network of virulence in *A. citrulli* .

## 4 Experimental Procedures

### 4.1 Bacterial Strains, Plasmids, Growth Conditions, and Plants Material

The bacterial strains and plasmids used in this study are listed in Table S1. *A. citrulli* strains were grown at 28°C in nutrient‐rich LB medium (Sambrook et al. 1982) or nutrient‐poor XVM2 medium (Wengelnik et al. 1996). *E. coli* strains were cultured at 37°C in LB medium. When required, antibiotics were added at the following final concentrations: rifamycin (Rif) 100 mg/mL, kanamycin (Km) 50 mg/mL, gentamicin (Gm) 50 mg/mL. Melon (cv. Huanghou) and *N. benthamiana* were planted for pathogenicity and HR tests, respectively.

### 4.2 Construction of Bacterial Mutants and Genetic Complementation

The markerless deletion mutants were constructed with the homologous double‐crossover method as described previously (Cheng et al. 2019) with some modifications. The upstream and downstream fragment of targeted gene were amplified with the primers listed in Table S2. The primers were designed based on the genome of group II type strain AAC00‐1 (NC_008752.1). The purified DNA fragments were ligated with linearised suicide vector pK18*mobsacB*. The recombinant vector vectors pK18‐*1583* and pK18‐*1584* was transferred from *E. coli* BW20676 into the wild‐type xjL12 by biparental mating. The mutants were screened on LB plates supplemented with 10% sucrose.

For construction of complemented strains, the full‐length fragments of the *envZ*_*Ac*_, *ompR*_*Ac*_, and *ompR*_*Ac*_ */envZ*_*Ac*_ operon were generated by PCR with primers listed in Table S2 and ligated into the broad‐host vector pBBR1MCS‐5. Recombinant *envZ*_*Ac*_^*H266A*^ and *ompR*_*Ac*_^*D59A*^, where putative phosphorylation sites were replaced by Ala, were generated by overlap PCR. Finally, the recombinant vectors were introduced into corresponding strains by biparental mating as described above.

### 4.3 Pathogenicity Assays

The protocols for investigating *A. citrulli* virulence were described in our prior publication (Wang, Zhao, et al. 2021). The severity of the disease was quantified by the disease index (DI), which was calculated as previously described (Wang, Zhao, et al. 2021).

### 4.4 Measurement of Growth Curves

The bacterial suspensions at an OD_600_ of 1.0 were added to LB with different pH or concentrations of NaCl at a ratio of 1:100 (vol/vol). The cell turbidity at a certain time was measured by spectrophotometer (BioPhotometer; Eppendorf). The final cell turbidity is the average of three replicate experiments.

### 4.5 Characterisation of Virulence‐Related Phenotypes

Swimming motility assay was performed in LB with 0.3% agar as previously described (Wang et al. 2016). The crystal violet assay was performed to measure biofilm formation in LB or XVM2 medium within 24‐well polyvinyl chloride plates as detailed before (Wang, Zhao, et al. 2021). The Congo red assay was carried out as described previously with some modifications (Xie et al. 2019). LB containing 1% agar was supplemented with Congo red dye (0.4 mg/mL) sterilised by filtration. Cell suspension at OD_600_ of 1.0 was spotted onto the Congo red plates. The colony morphology was photographed after incubation for 4–5 days.

The HR assay was conducted as described previously (Wang, Zhao, et al. 2021). The accumulation of ROS in the syringe‐injected region was determined with 3,3′‐diaminobenzidine (DAB) staining as described previously with some modifications (Daudi and O'Brien 2012). Briefly, the inoculated *N. benthamiana* leaves were sampled and immersed in DAB solution (1 mg/mL, pH 3.5), and then washed with distilled water. The stained leaves were decolourised by boiling in ethanol for 20 min. In these assays, each phenotype was determined three times, and at least three replicates for each strain were performed per experiment.

### 4.6 Nanoluciferase Activity Assay

A Nanoluciferase (*nluc*) reporter gene was used to determine the activity of the promoter as described previously (Xie et al. 2023) with minor modifications. The reporter plasmids pBBR‐P*ompR*_*Ac*_‐*nluc* and pBBR‐P*hrpG*‐*nluc* were introduced to WT strain or mutants. The detection of Nanoluciferase activity was conducted according to manufacturer's instructions (Nano‐Glo Luciferase Assay System; Promega). The OD_600_‐normalised cellular luminescence was taken as the activity of the promoter.

### 4.7 Protein Expression and Purification

His_6_‐tagged recombinant proteins OmpR_Ac_, C‐terminal EnvZ_Ac_ (EnvZ_Ac_C, amino acids 200–505) lacking putative transmembrane domain, and their derivative OmpR_Ac_^D59A^, EnvZ_Ac_C^H266A^ were expressed in *E. coli* BL21 (DE3) by corresponding recombinant pET30a, as detailed before (Shao et al. 2021). The open reading frames (ORF) of OmpR_Ac_ and EnvZ_Ac_C were amplified with the primers listed in Table S2. The OmpR_Ac_^D59A^ and EnvZ_Ac_C^H266A^ ORFs were generated by overlap PCR.

### 4.8 Phosphorylation of OmpR_Ac_

The phosphorylation reaction was carried out in phosphorylation buffer (50 mM Tris–HCl, pH 7.8, 25 mM NaCl, 25 mM KCl, 5 mM MgCl_2_, 2 mM dithiothreitol [DTT]) as described previously (Deng et al. 2014). The purified EnvZ_Ac_C (1 μM) and OmpR_Ac_ (20 μM) proteins were incubated with 20 mM ATP in reaction buffer at room temperature for 30 min to generate phosphorylated OmpR_Ac_.

### 4.9 EMSA

EMSA was performed as described previously (Xie et al. 2019). DNA probes were amplified through PCR with primers listed in Table S2. Various amounts of proteins were mixed with probes (50 ng) in a total volume of 20 μL. The protein–DNA mixture was incubated in EMSA binding buffer (10 mM Tris–HCl, pH 7.4, 50 mM KCl, 50 mM MgCl_2_, 5% glycerol) at room temperature for 20 min and analysed by 6% native polyacrylamide gel electrophoresis in 0.5 × TBE (Tris/boric acid/EDTA) running buffer (90 V, 90 min). The gels were stained by GelRed dye for 20 min. The bands were visualised and photographed by a gel imaging system GenoSens 2000 (Clinx). This assay was repeated at least three times.

### 4.10 MST Assay

The MST assay for investigating the interaction between OmpR_Ac_ and the *hrpG* promoter (P*hrpG*) was performed using a Monolith NT.115 MicroScale Thermophoresis instrument (NanoTemper Technologies). P*hrpG* was labelled by a far‐red‐fluorescent Cyanine5 (Cy5) at the 5′ end and acted as target. Serial dilutions of OmpR_Ac_ or OmpR_Ac_^D59A^ (0.6 nM to 10 μM) and Cy5‐labelled P*hrpG*, which was constant at 10 nM, were mixed in MST buffer (50 mM Tris–HCl, pH 7.4, 150 mM NaCl, 10 mM MgCl_2_, 0.05% Tween 20). The reaction mixtures were then loaded into the Monolith NT.115 capillaries and measured at medium MST power. Finally, MO.Analysis software (v. 2.3) was used for fitting the curve and calculating the dissociation constant (*K*_d_).

### 4.11 RT‐qPCR

RT‐qPCR analysis was carried out exactly as detailed in previous study (Wang, Zhao, et al. 2021). For detecting T3SS genes, strains were transferred to XVM2 medium to induce T3SS. For detecting the *omR*_*Ac*_ */envZ*_*Ac*_ operon in response to stressors, strains were transferred to high osmolality or acidic/alkaline medium. The primers for RT‐qPCR are listed in Table S2. This analysis was repeated independently three times and each sample carried out three technical repeats per experiment.

### 4.12 Transmission Electron Microscopy

Fresh colonies on LB plates with 1% agar were gently resuspended with distilled water. Bacterial suspensions were transferred onto the copper grid and then negatively stained with phosphotungstic acid. Bacterial flagella were observed and photographed with a transmission electron microscope HT7800 (Hitachi) at an operating voltage of 80 kV. The lengths of flagella were measured by ImageJ software.

### 4.13 Statistical Analysis

All statistical analyses and graph plotting were conducted by the Graphpad Prism software (v. 9.5).

## Author Contributions

Y.W. and Y.T. designed the experiments. Y.W., C.S., L.C., S.W. and W.C. performed the experiments and analysed the data. Y.W. and Y.T. wrote the manuscript. Y.T., B.H. and R.W. revised the manuscript and provided guidance for the experiments. All authors contributed to the article and approved the submitted version.

## Conflicts of Interest

The authors declare no conflicts of interest.

## Supporting information


**Figure S1.** Screening a transposon‐insertion library by inoculating *Nicotiana benthamiana* leaves. Wild‐type (WT) strain xjL12 (left) and Tn*5*‐insertion mutant (right) were adjusted to OD_600_ of 0.3 with sterile water and were injected into *N. benthamiana*. The image was acquired at 48 h post‐inoculation. Each strain was subjected to the experiment in triplicate.

**Figure S2.** Sequence analysis of Tn*5*‐insertion fragment in *Acidovorax citrulli* xjL12. (a) Schematic representation of the Tn*5* insertion and sequencing with primer pair Tn5‐F/Tn5‐R. (b) Tn*5*‐insertion fragment was obtained by Sanger sequencing with Tn5‐F/Tn5‐R shaded with yellow. Bases in blue and in red indicate the nucleotides of transposon delivery vector pUTKm DNA and its flanking region, respectively. The red nucleotides are identified as the part of *Aave_1583* by BlastN.

**Figure S3.** Reverse transcription (RT)‐PCR and phylogenetic analysis of *Aave_1584‐Aave_1583*. (a) Validation of the operon *Aave_1584‐Aave_1583* by RT‐PCR. Arrows above genes were primers used to investigate the operon structure. DNA was the total genome extracted from *Acidovorax citrulli* xjL12. cDNA was reverse transcribed from total RNA. PCR with primer pair F3R3 served as positive control, while primer F1R1 served as negative control. M, marker 2000 bp. (b, c) Phylogenetic tree (right) and secondary structures (left) of 15 OmpRs (b) and EnvZs (c) from diverse bacteria. Phylogenetic tree was inferred using the neighbour‐joining method and constructed with MEGA X. The branch lengths represent evolutionary distances computed using the Poisson correction method. The percentage of replicate trees in which the associated taxa clustered together in the bootstrap test (2000 replicates) are shown next to the branches. The branches harbouring *A. citrulli* spp. are highlighted with red. *A. citrulli* strains are shaded with yellow. The schematic diagram of domains predicted by SMART was generated by TBtools‐II.

**Figure S4.** Reverse transcription‐quantitative PCR analysis of the expression of *ompR*_*Ac*_ and *envZ*_*Ac*_ (a–c), and flagellum‐related genes (d). (a–c) The total RNA of xjL12, subjected to osmotic stress with different concentrations of NaCl (a), acidic/alkaline stress (b), and to poor nutrition medium XVM2 (c) were extracted after incubation for 4 h. (d) The total RNA was extracted from wild‐type (WT), Δ*envZ*_*Ac*_, Δ*ompR*_*Ac*_ */envZ*_*Ac*_, and complemented strain C*envZ*_*Ac*_, C*ompR*_*Ac*_ */envZ*_*Ac*_, which were incubated in Luria Bertani medium to OD_600_ = 1.0. Total RNA samples were reverse transcribed to cDNA for following qPCR. Error bars represent mean standard deviation of three replicates. Asterisks indicate significant differences compared with the WT strain (Student’s *t* test, *p < 0.05).

**Figure S5.** Electrophoretic mobility shift assay of OmpR_Ac_ with the promoter region of *ompR*_*Ac*_ (a), OmpRAc^D59A^ with the promoter region of *hrpG* (b). The OmpR_Ac_ or OmpRAc^D59A^ (20 μm) was phosphorylated by EnvZ_Ac_C (1 μm) in phosphorylation buffer. The various amounts of OmpR_Ac_ and OmpRAc^D59A^ was incubated with its promoter (P*ompR*_*Ac*_, 50 ng) and *hrpG* promoter (P*hrpG*, 50 ng) in binding buffer for 20 min at room temperature. The reaction mixture was analysed by 6% polyacrylamide gel electrophoresis. The gel was stained with GelRed dye and photographed by a gel imaging system. The OmpR_Ac_ incubated with EnvZ_Ac_C^H266A^ incapable of autophosphorylation acted as a negative control. This assay was repeated three times.

**Figure S6.** The promoter activity of *ompR*_*Ac*_ in XVM2 with elevated osmotic (a) and acidic/alkaline pressure (b). Wild‐type strain xjL12 harbouring pBBR‐P*ompR*_*Ac*_‐*nluc* was cultured under different conditions with an initial concentration of OD_600_ = 0.5. P*ompR*_*Ac*_‐*nluc* was detected after 4 h of cultivation. Data represent three biological replicates and were analysed by Student’s *t* test (**p < 0.01). Error bars represent mean standard deviation.

**Figure S7.** Effect of OmpR_Ac_/EnvZ_Ac_ on *Acidovorax citrulli* xjL12 growth in Luria Bertani (LB) medium (a), the sensitivity to high temperature (b) and H_2_O_2_ (c). (a) The in‐frame deletions of OmpR_Ac_ or EnvZ_Ac_ were inoculated to LB medium to examine the growth rate. (b) *A. citrulli* strains cultured overnight were adjusted to OD_600_ = 0.1 with fresh LB. Cell suspensions were incubated in water bath with 50°C for 0, 3 and 6 min, and then transferred to a 28°C shaker for 8 h. The final cell concentrations were measured at OD_600_. (c) *A. citrulli* strains cultured overnight were adjusted to OD_600_ = 0.3 with fresh LB. LB agar was supplemented with cell suspensions at a volume ratio of 1:50. A paper disk diameter was placed the centre of each plate containing bacteria. Five microlitres of 10% H_2_O_2_ was dropped on the paper disks. The diameters of inhibition zones were measured after incubation for 48 h at 28°C. Data represent three biological replicates and were analysed using the Student’s *t* test (ns, no significance). Error bars represent mean standard deviation. WT, wild‐type strain xjL12; Δ*envZ*_*Ac*_, Δ*ompR*_*Ac*_ and Δ*ompR*_*Ac*_/*envZ*_*Ac*_, the in‐frame deletion of *envZ*_*Ac*_, *ompR*_*Ac*_ and both *ompR*_*Ac*_/*envZ*_*Ac*_, respectively. C*envZ*_*Ac*_, C*ompR*_*Ac*_ and C*ompR*_*Ac*_/*envZ*_*Ac*_: complemented strain of Δ*envZ*_*Ac*_, Δ*ompR*_*Ac*_ and Δ*ompR*_*Ac*_ /*envZ*_*Ac*_, respectively.

**Figure S8.** The population of bacteria in true leaves (a) and seeds (b). (a) True leaves of melon plants were inoculated with cell suspensions (OD_600_ = 0.3) by spraying and sampled at 2 and 4 days post‐inoculation (dpi). Disks (8 mm) were collected from leaves and triturated to population quantification. (b) Each melon seed was inoculated with 5 μL of cell suspension (approximately 10^6^ CFU/mL). Inoculated seeds were maintained on moistened paper at 28°C. Bacterial populations within inoculated seeds were quantified at 2 and 4 dpi. The error bars represent the standard deviation of three replicates. Asterisks indicate significant differences compared with the wild‐type (WT) strain at different sampling time points (Student’s *t* test, *p < 0.05, **p < 0.01; ns, no significance). WT‐EV: wild‐type strain xjL12 harbouring the empty vector pBBR1MCS‐5 (EV); Δ*envZ*_*Ac*_‐EV, Δ*ompR*_*Ac*_‐EV and Δ*ompR*_*Ac*_ /*envZ*_*Ac*_ ‐EV: *envZ*_*Ac*_, *ompR*_*Ac*_ single mutant and dual mutant strain harbouring the empty vector pBBR1MCS‐5, respectively; C*envZ*_*Ac*_, C*ompR*_*Ac*_ and C*ompR*_*Ac*_/*envZ*_*Ac*_: complemented strain of Δ*envZ*_*Ac*_, Δ*ompR*_*Ac*_ and Δ*ompR*_*Ac*_ /*envZ*_*Ac*_, respectively.

**Figure S9.** Biofilm formation of tested strains in Luria Bertani medium (LB) (a) and minimal medium XVM2 (b). The tested strains were adjusted to an OD_600_ of 1.0 with corresponding fresh medium. 20 μL of cell suspensions were added to 2 mL LB or XVM2 in each well of 24‐well polyvinyl chloride plates. After 48 h, the medium was removed, and the plate was dried at 80°C for 20 min following the wells rinsed with distilled water. The biofilm was stained by crystal violet and photographed. The crystal violet was solubilised with ethanol and measured at OD_590_. The error bars represent the standard deviation of the means from three independent experiments. Asterisks indicate significant differences compared with the wild‐type (WT) strain (Student’s *t* test, **p < 0.01, ***p < 0.001; ns, no significance). WT‐EV: wild‐type strain xjL12 harbouring the empty vector pBBR1MCS‐5 (EV); Δ*envZ*_*Ac*_‐EV, Δ*ompR*_*Ac*_‐EV and Δ*ompR*_*Ac*_ /*envZ*_*Ac*_ ‐EV: *envZ*_*Ac*_, *ompR*_*Ac*_ single mutant and dual mutant strain harbouring the empty vector pBBR1MCS‐5, respectively; C*envZ*_*Ac*_, C*ompR*_*Ac*_ and C*ompR*_*Ac*_/*envZ*_*Ac*_: complemented strain of Δ*envZ*_*Ac*_, Δ*ompR*_*Ac*_ and Δ*ompR*_*Ac*_ /*envZ*_*Ac*_, respectively; *envZ*_*Ac*_^H266A^: *envZ*_*Ac*_ point mutation with the His266 substituted by Ala; *ompR*_*Ac*_^D59A^: *ompR*_*Ac*_ point mutation with the Asp59 substituted by Ala.

**Figure S10.** The original images of Congo red staining (a) and swimming motility (b). WT‐EV: wild‐type strain xjL12 harbouring the empty vector pBBR1MCS‐5 (EV); Δ*envZ*_*Ac*_‐EV, Δ*ompR*_*Ac*_‐EV and Δ*ompR*_*Ac*_ /*envZ*_*Ac*_ ‐EV: *envZ*_*Ac*_, *ompR*_*Ac*_ single mutant and dual mutant strain harbouring the empty vector pBBR1MCS‐5, respectively; C*envZ*_*Ac*_, C*ompR*_*Ac*_ and C*ompR*_*Ac*_/*envZ*_*Ac*_: complemented strain of Δ*envZ*_*Ac*_, Δ*ompR*_*Ac*_ and Δ*ompR*_*Ac*_ /*envZ*_*Ac*_, respectively; *envZ*_*Ac*_^H266A^: *envZ*_*Ac*_ point mutation with the His266 substituted by Ala; *ompR*_*Ac*_^D59A^: *ompR*_*Ac*_ point mutation with the Asp59 substituted by Ala.

Table S1.

Table S2.

## Data Availability

The data that supports the findings of this study are available in the Supporting Information of this article.
