# Supplementary figures and images for: Osmotic and pH Stress‐Responsive Two‐Component System, OmpR/EnvZ, Modulates Type III Secretion, Biofilm Formation, Swimming Motility and Virulence in Acidovorax citrulli xjL12
Source: Mol Plant Pathol. 2025 Jun 16;26(6):e70107. doi: 10.1111/mpp.70107 (PMC12170943; doi:10.1111/mpp.70107)

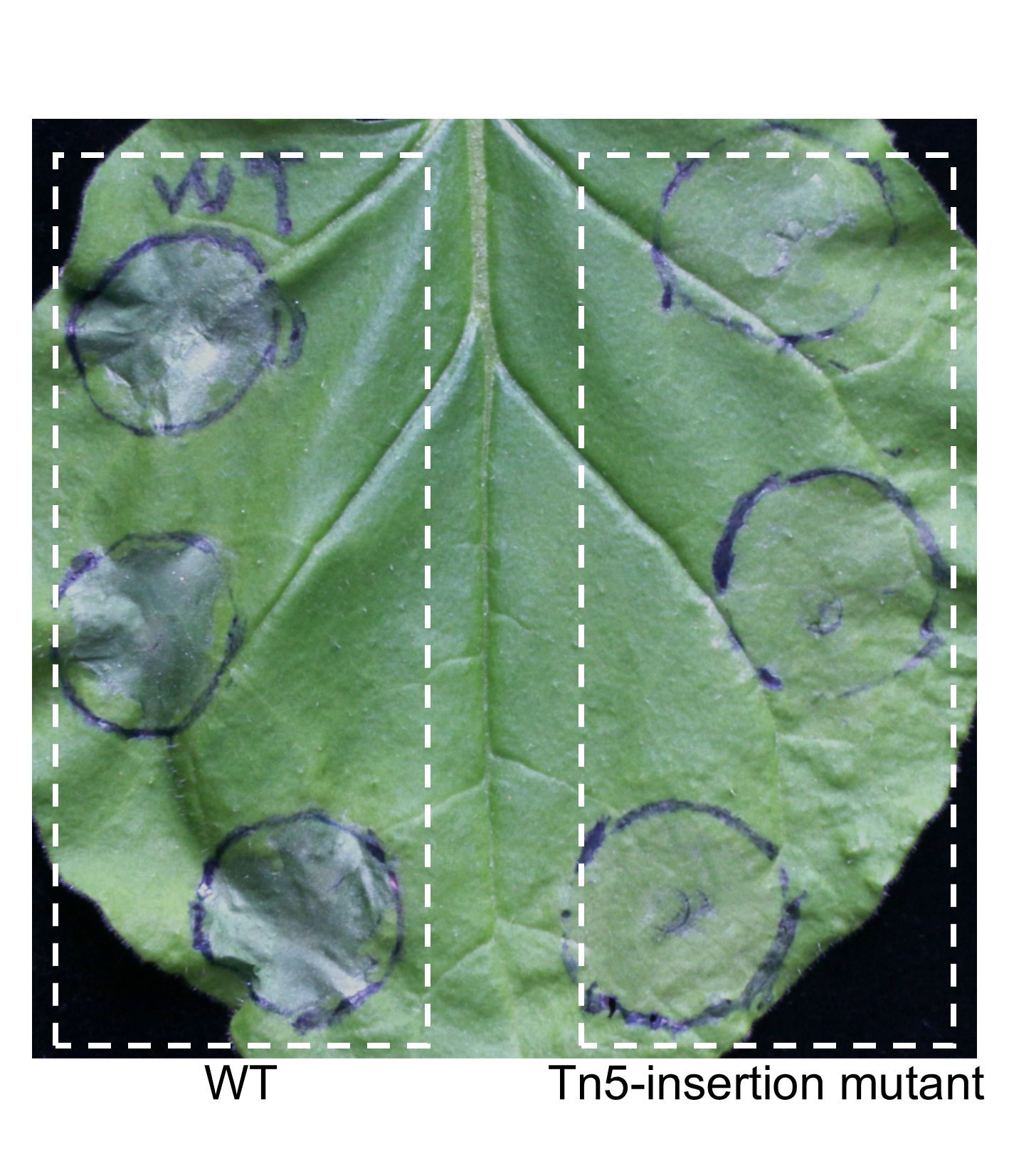

Supplement: Supplementary file 1 — Figure S1. Screening a transposon‐insertion library by inoculating Nicotiana benthamiana leaves. Wild‐type (WT) strain xjL12 (left) and Tn5‐insertion mutant (right) were adjusted to OD600 of 0.3 with sterile water and were injected into N. benthamiana. The image was acquired at 48 h post‐inoculation. Each strain was subjected to the experiment in triplicate. [file MPP-26-e70107-s001.tif]

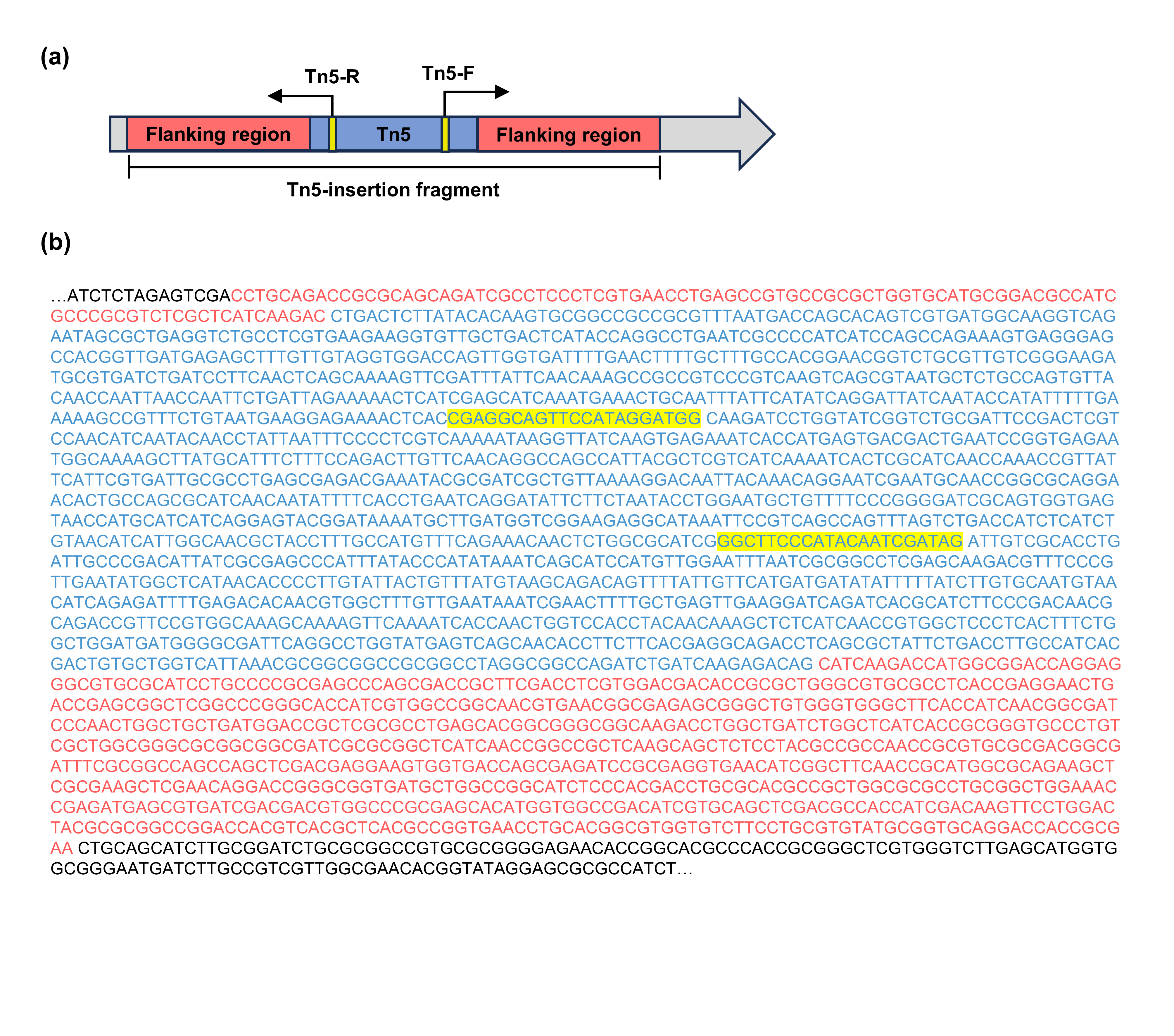

Supplement: Supplementary file 2 — Figure S2. Sequence analysis of Tn5‐insertion fragment in Acidovorax citrulli xjL12. (a) Schematic representation of the Tn5 insertion and sequencing with primer pair Tn5‐F/Tn5‐R. (b) Tn5‐insertion fragment was obtained by Sanger sequencing with Tn5‐F/Tn5‐R shaded with yellow. Bases in blue and in red indicate the nucleotides of transposon delivery vector pUTKm DNA and its flanking region, respectively. The red nucleotides are identified as the part of Aave_1583 by BlastN. [file MPP-26-e70107-s007.tif]

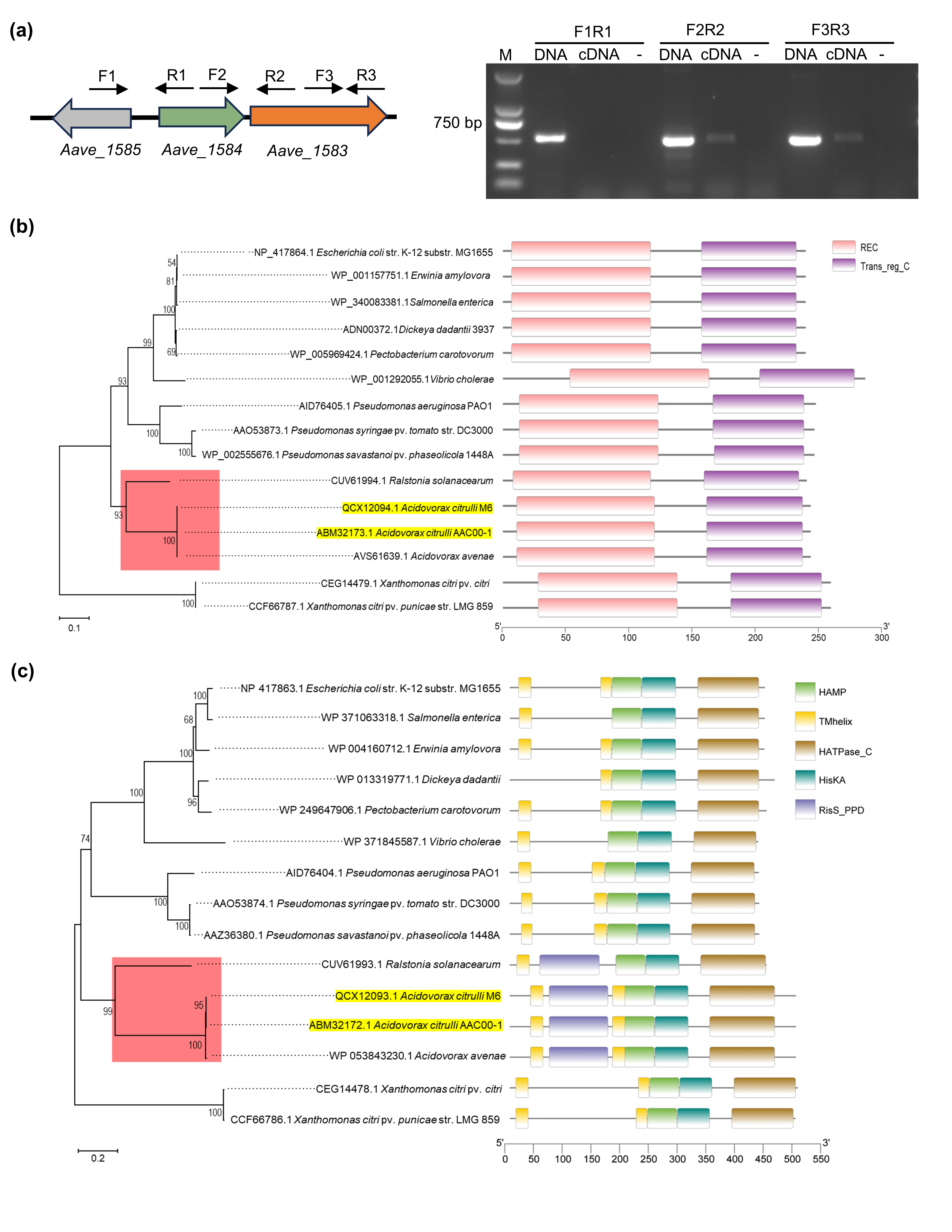

Supplement: Supplementary file 3 — Figure S3. Reverse transcription (RT)‐PCR and phylogenetic analysis of Aave_1584‐Aave_1583. (a) Validation of the operon Aave_1584‐Aave_1583 by RT‐PCR. Arrows above genes were primers used to investigate the operon structure. DNA was the total genome extracted from Acidovorax citrulli xjL12. cDNA was reverse transcribed from total RNA. PCR with primer pair F3R3 served as positive control, while primer F1R1 served as negative control. M, marker 2000 bp. (b, c) Phylogenetic tree (right) and secondary structures (left) of 15 OmpRs (b) and EnvZs (c) from diverse bacteria. Phylogenetic tree was inferred using the neighbour‐joining method and constructed with MEGA X. The branch lengths represent evolutionary distances computed using the Poisson correction method. The percentage of replicate trees in which the associated taxa clustered together in the bootstrap test (2000 replicates) are shown next to the branches. The branches harbouring A. citrulli spp. are highlighted with red. A. citrulli strains are shaded with yellow. The schematic diagram of domains predicted by SMART was generated by TBtools‐II. [file MPP-26-e70107-s002.tif]

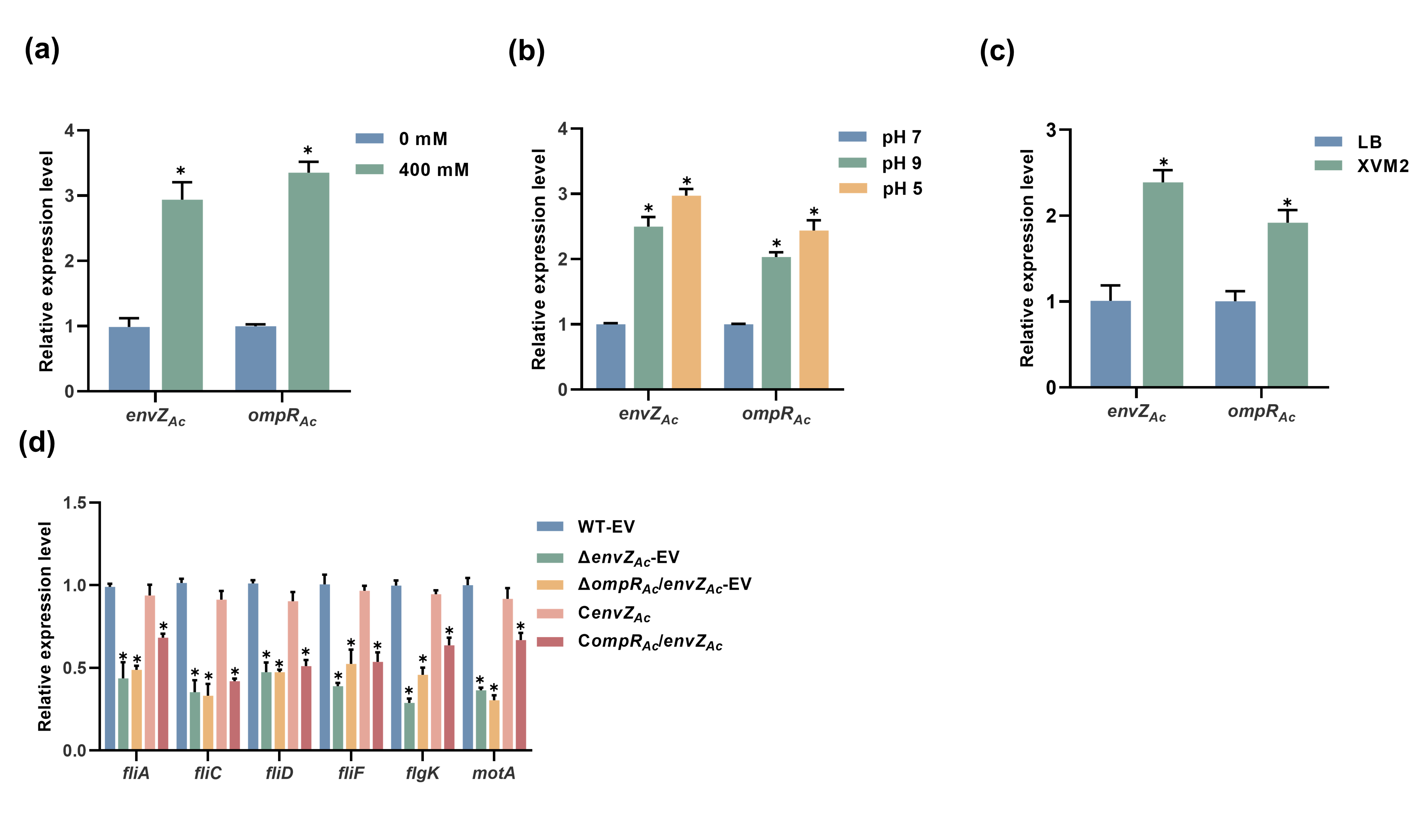

Supplement: Supplementary file 4 — Figure S4. Reverse transcription‐quantitative PCR analysis of the expression of ompR Ac and envZ Ac (a–c), and flagellum‐related genes (d). (a–c) The total RNA of xjL12, subjected to osmotic stress with different concentrations of NaCl (a), acidic/alkaline stress (b), and to poor nutrition medium XVM2 (c) were extracted after incubation for 4 h. (d) The total RNA was extracted from wild‐type (WT), ΔenvZ Ac , ΔompR Ac /envZ Ac , and complemented strain CenvZ Ac , CompR Ac /envZ Ac , which were incubated in Luria Bertani medium to OD600 = 1.0. Total RNA samples were reverse transcribed to cDNA for following qPCR. Error bars represent mean standard deviation of three replicates. Asterisks indicate significant differences compared with the WT strain (Student’s t test, *p < 0.05). [file MPP-26-e70107-s003.tif]

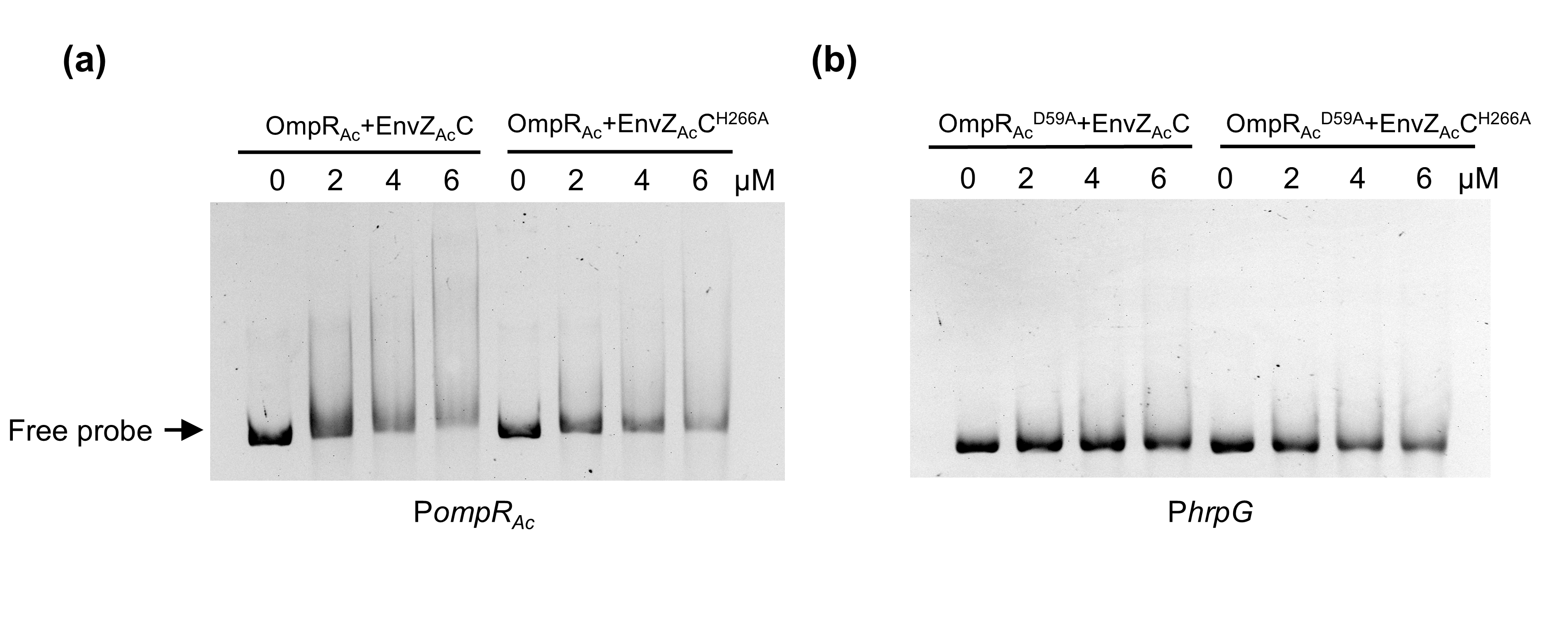

Supplement: Supplementary file 5 — Figure S5. Electrophoretic mobility shift assay of OmpRAc with the promoter region of ompR Ac (a), OmpRAcD59A with the promoter region of hrpG (b). The OmpRAc or OmpRAcD59A (20 μm) was phosphorylated by EnvZAcC (1 μm) in phosphorylation buffer. The various amounts of OmpRAc and OmpRAcD59A was incubated with its promoter (PompR Ac , 50 ng) and hrpG promoter (PhrpG, 50 ng) in binding buffer for 20 min at room temperature. The reaction mixture was analysed by 6% polyacrylamide gel electrophoresis. The gel was stained with GelRed dye and photographed by a gel imaging system. The OmpRAc incubated with EnvZAcCH266A incapable of autophosphorylation acted as a negative control. This assay was repeated three times. [file MPP-26-e70107-s009.tif]

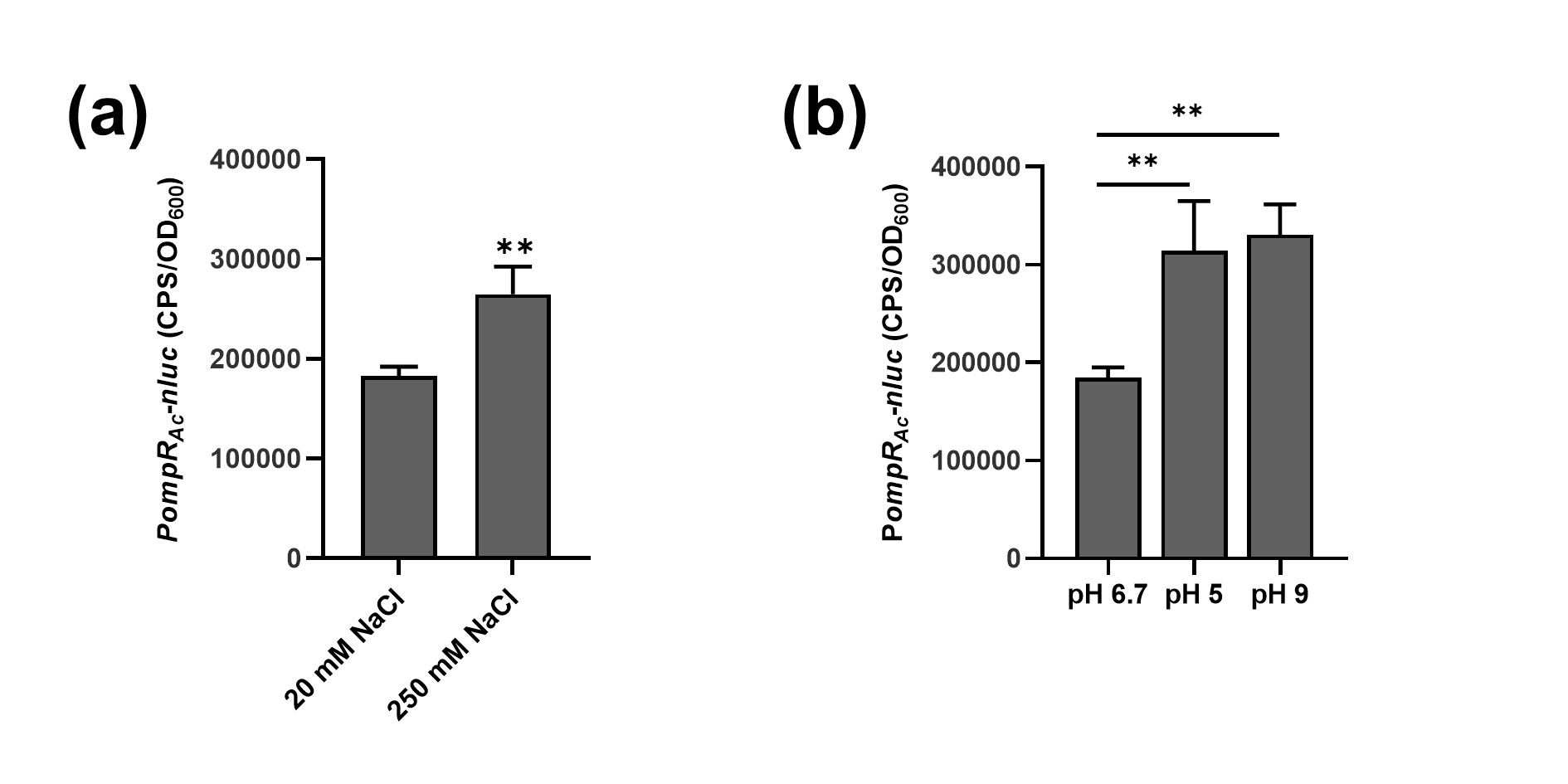

Supplement: Supplementary file 6 — Figure S6. The promoter activity of ompR Ac in XVM2 with elevated osmotic (a) and acidic/alkaline pressure (b). Wild‐type strain xjL12 harbouring pBBR‐PompR Ac ‐nluc was cultured under different conditions with an initial concentration of OD600 = 0.5. PompR Ac ‐nluc was detected after 4 h of cultivation. Data represent three biological replicates and were analysed by Student’s t test (**p < 0.01). Error bars represent mean standard deviation. [file MPP-26-e70107-s008.tif]

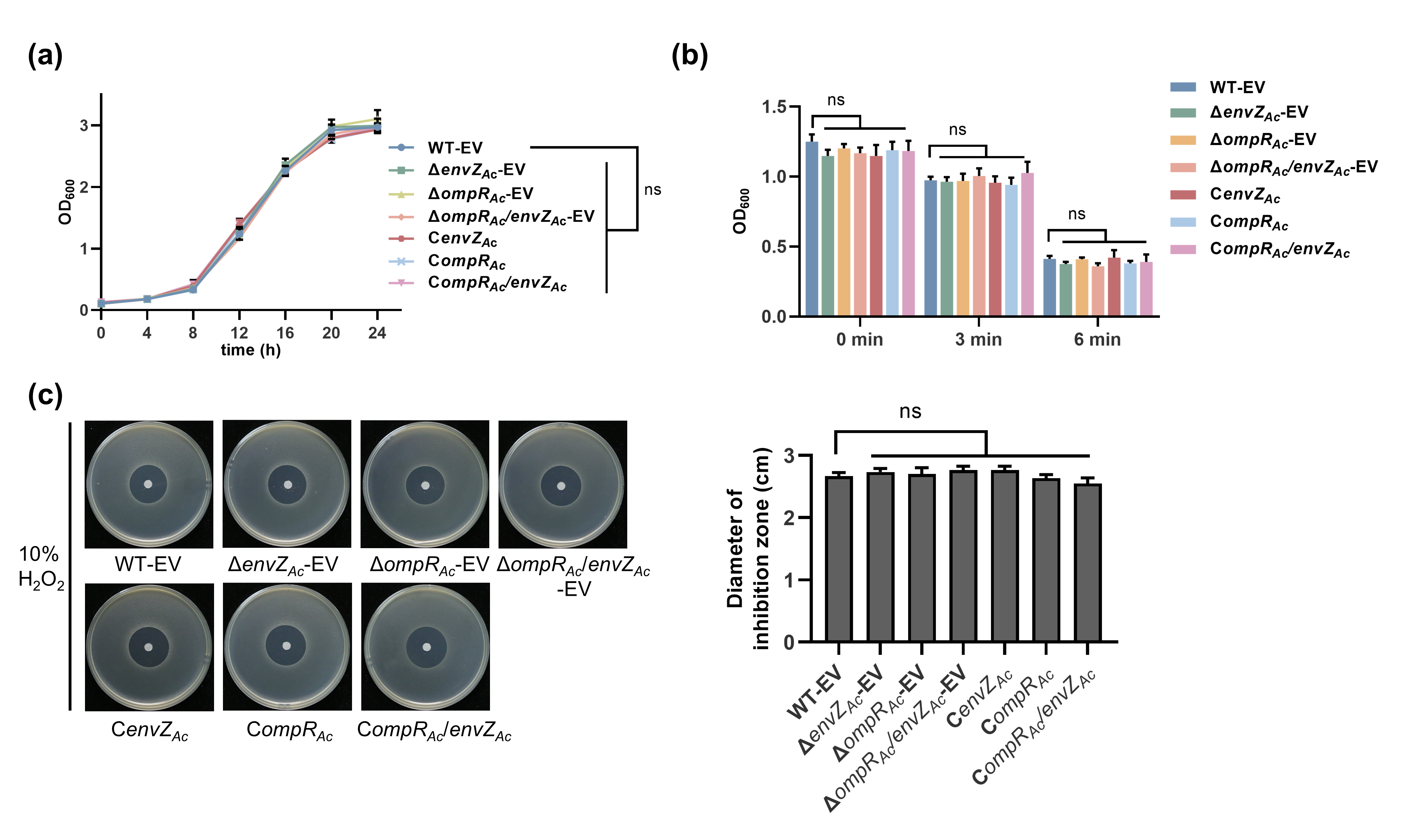

Supplement: Supplementary file 7 — Figure S7. Effect of OmpRAc/EnvZAc on Acidovorax citrulli xjL12 growth in Luria Bertani (LB) medium (a), the sensitivity to high temperature (b) and H2O2 (c). (a) The in‐frame deletions of OmpRAc or EnvZAc were inoculated to LB medium to examine the growth rate. (b) A. citrulli strains cultured overnight were adjusted to OD600 = 0.1 with fresh LB. Cell suspensions were incubated in water bath with 50°C for 0, 3 and 6 min, and then transferred to a 28°C shaker for 8 h. The final cell concentrations were measured at OD600. (c) A. citrulli strains cultured overnight were adjusted to OD600 = 0.3 with fresh LB. LB agar was supplemented with cell suspensions at a volume ratio of 1:50. A paper disk diameter was placed the centre of each plate containing bacteria. Five microlitres of 10% H2O2 was dropped on the paper disks. The diameters of inhibition zones were measured after incubation for 48 h at 28°C. Data represent three biological replicates and were analysed using the Student’s t test (ns, no significance). Error bars represent mean standard deviation. WT, wild‐type strain xjL12; ΔenvZ Ac , ΔompR Ac and ΔompR Ac /envZ Ac , the in‐frame deletion of envZ Ac , ompR Ac and both ompR Ac /envZ Ac , respectively. CenvZ Ac , CompR Ac and CompR Ac /envZ Ac : complemented strain of ΔenvZ Ac , ΔompR Ac and ΔompR Ac /envZ Ac , respectively. [file MPP-26-e70107-s004.tif]

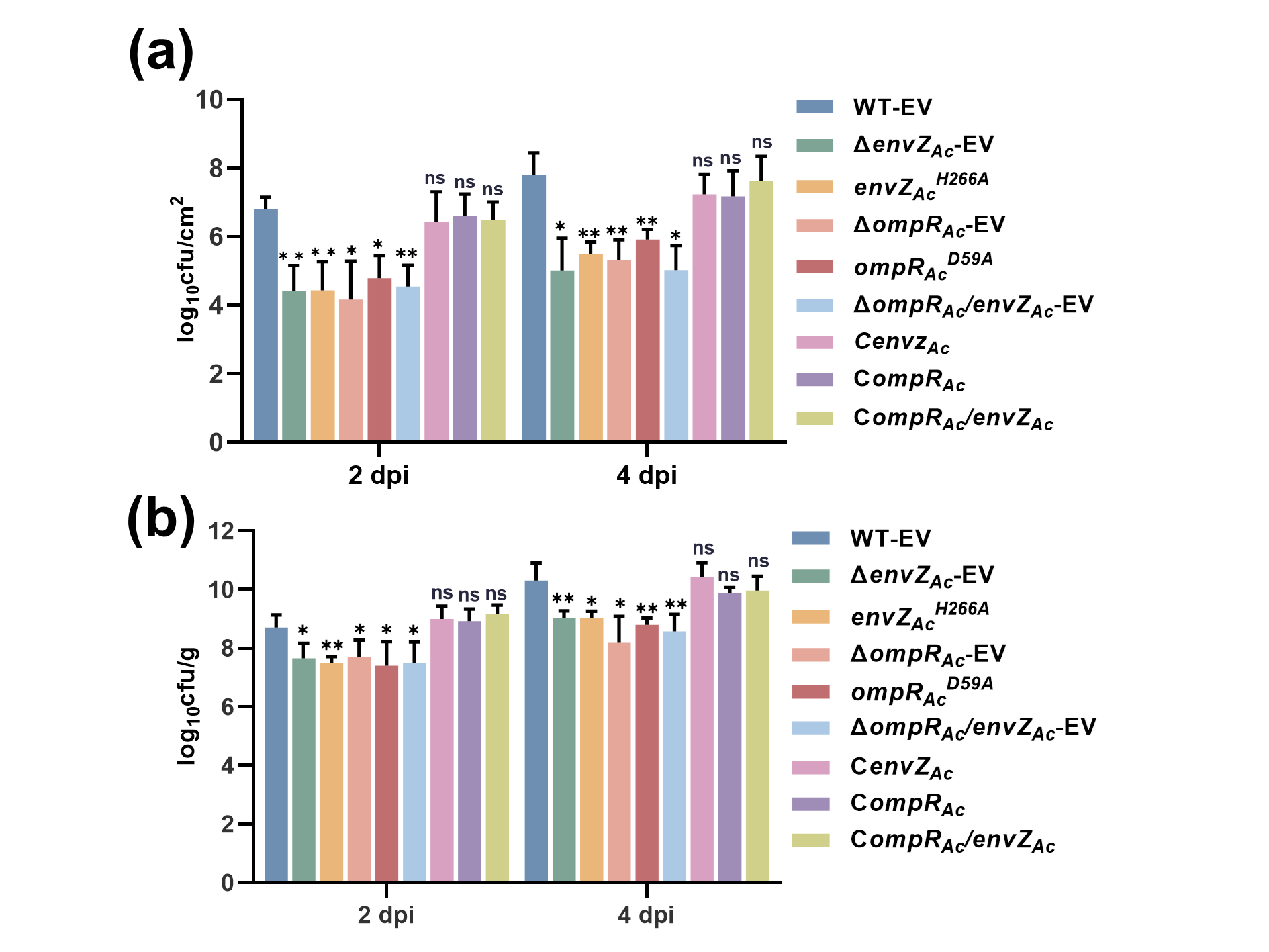

Supplement: Supplementary file 8 — Figure S8. The population of bacteria in true leaves (a) and seeds (b). (a) True leaves of melon plants were inoculated with cell suspensions (OD600 = 0.3) by spraying and sampled at 2 and 4 days post‐inoculation (dpi). Disks (8 mm) were collected from leaves and triturated to population quantification. (b) Each melon seed was inoculated with 5 μL of cell suspension (approximately 106 CFU/mL). Inoculated seeds were maintained on moistened paper at 28°C. Bacterial populations within inoculated seeds were quantified at 2 and 4 dpi. The error bars represent the standard deviation of three replicates. Asterisks indicate significant differences compared with the wild‐type (WT) strain at different sampling time points (Student’s t test, *p < 0.05, **p < 0.01; ns, no significance). WT‐EV: wild‐type strain xjL12 harbouring the empty vector pBBR1MCS‐5 (EV); ΔenvZ Ac ‐EV, ΔompR Ac ‐EV and ΔompR Ac /envZ Ac ‐EV: envZ Ac , ompR Ac single mutant and dual mutant strain harbouring the empty vector pBBR1MCS‐5, respectively; CenvZ Ac , CompR Ac and CompR Ac /envZ Ac : complemented strain of ΔenvZ Ac , ΔompR Ac and ΔompR Ac /envZ Ac , respectively. [file MPP-26-e70107-s012.tif]

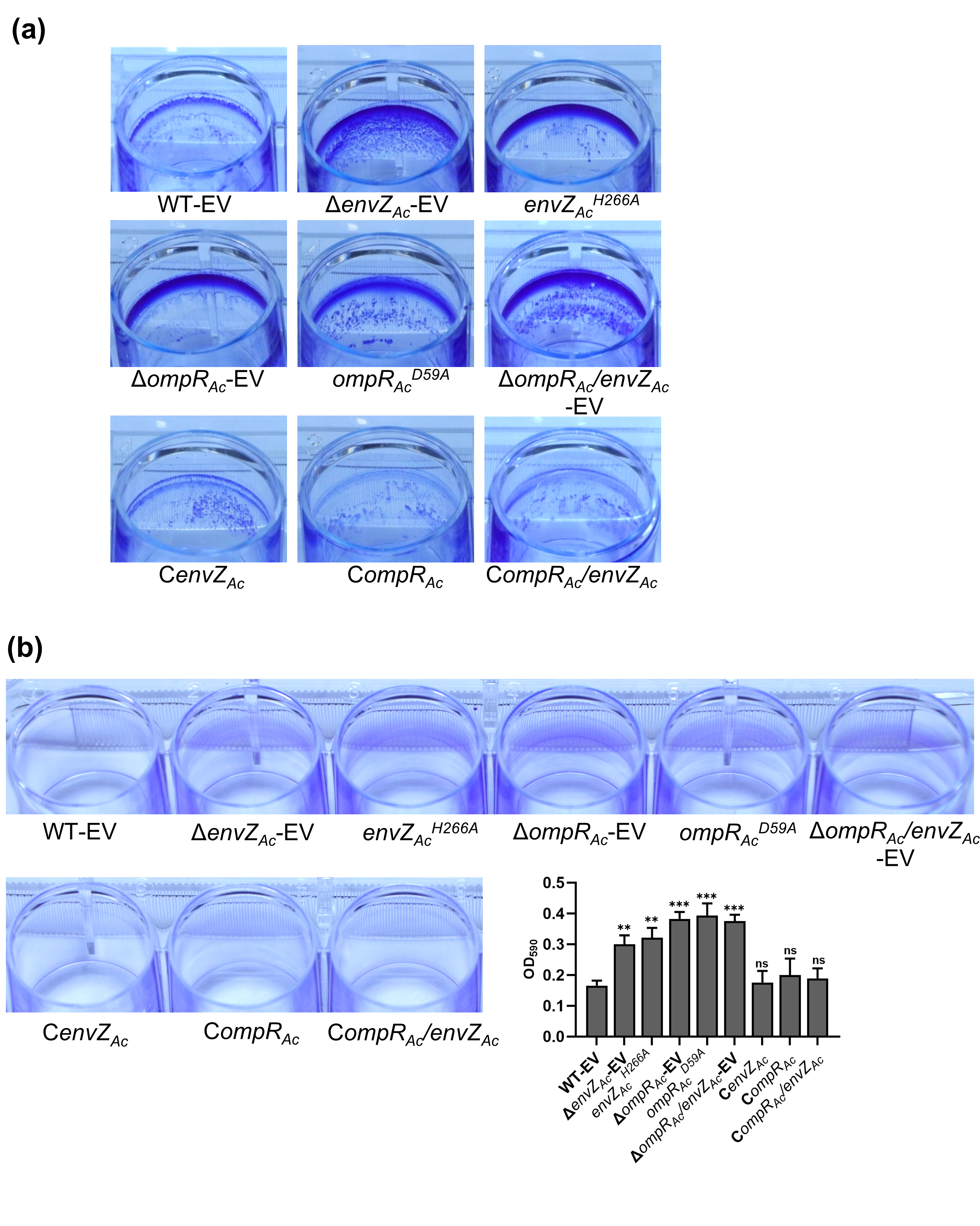

Supplement: Supplementary file 9 — Figure S9. Biofilm formation of tested strains in Luria Bertani medium (LB) (a) and minimal medium XVM2 (b). The tested strains were adjusted to an OD600 of 1.0 with corresponding fresh medium. 20 μL of cell suspensions were added to 2 mL LB or XVM2 in each well of 24‐well polyvinyl chloride plates. After 48 h, the medium was removed, and the plate was dried at 80°C for 20 min following the wells rinsed with distilled water. The biofilm was stained by crystal violet and photographed. The crystal violet was solubilised with ethanol and measured at OD590. The error bars represent the standard deviation of the means from three independent experiments. Asterisks indicate significant differences compared with the wild‐type (WT) strain (Student’s t test, **p < 0.01, ***p < 0.001; ns, no significance). WT‐EV: wild‐type strain xjL12 harbouring the empty vector pBBR1MCS‐5 (EV); ΔenvZ Ac ‐EV, ΔompR Ac ‐EV and ΔompR Ac /envZ Ac ‐EV: envZ Ac , ompR Ac single mutant and dual mutant strain harbouring the empty vector pBBR1MCS‐5, respectively; CenvZ Ac , CompR Ac and CompR Ac /envZ Ac : complemented strain of ΔenvZ Ac , ΔompR Ac and ΔompR Ac /envZ Ac , respectively; envZ Ac H266A: envZ Ac point mutation with the His266 substituted by Ala; ompR Ac D59A: ompR Ac point mutation with the Asp59 substituted by Ala. [file MPP-26-e70107-s006.tif]

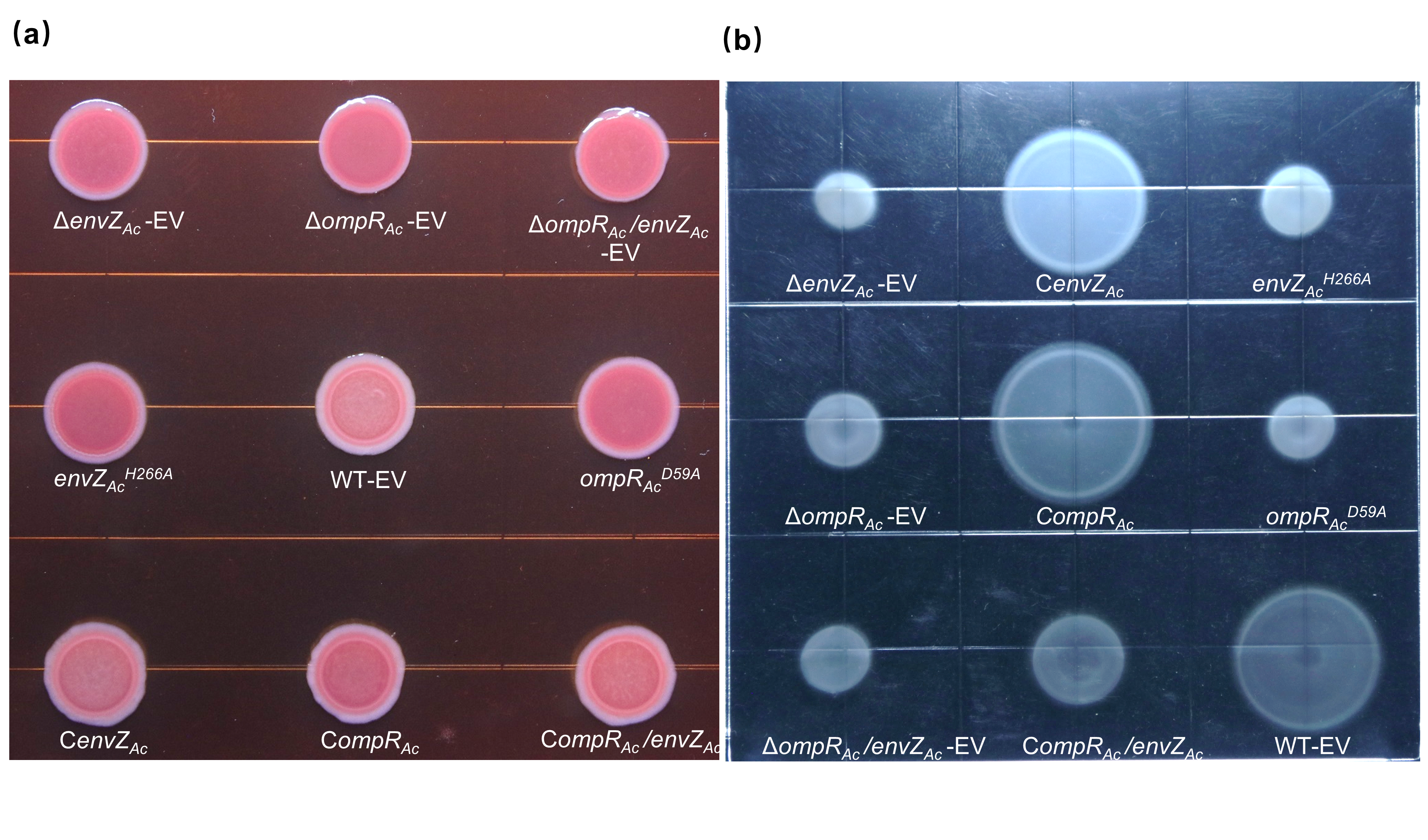

Supplement: Supplementary file 10 — Figure S10. The original images of Congo red staining (a) and swimming motility (b). WT‐EV: wild‐type strain xjL12 harbouring the empty vector pBBR1MCS‐5 (EV); ΔenvZ Ac ‐EV, ΔompR Ac ‐EV and ΔompR Ac /envZ Ac ‐EV: envZ Ac , ompR Ac single mutant and dual mutant strain harbouring the empty vector pBBR1MCS‐5, respectively; CenvZ Ac , CompR Ac and CompR Ac /envZ Ac : complemented strain of ΔenvZ Ac , ΔompR Ac and ΔompR Ac /envZ Ac , respectively; envZ Ac H266A: envZ Ac point mutation with the His266 substituted by Ala; ompR Ac D59A: ompR Ac point mutation with the Asp59 substituted by Ala. [file MPP-26-e70107-s010.tif]
