# Supplementary material for: Osmotic and pH Stress‐Responsive Two‐Component System, OmpR/EnvZ, Modulates Type III Secretion, Biofilm Formation, Swimming Motility and Virulence in Acidovorax citrulli xjL12
Source: Mol Plant Pathol. 2025 Jun 16;26(6):e70107. doi: 10.1111/mpp.70107 (PMC12170943; doi:10.1111/mpp.70107)
Supplement: Supplementary file 11 — Table S1. [file MPP-26-e70107-s005.docx]

**Table S1** Strains and plasmids used in this study

| **Strains and**  **plasmids** | **Genotype or description^a^** | **Source or**  **reference** |
| --- | --- | --- |
| **Strains** |  |  |
| *Acidovorax citrulli* |  |  |
| WT | Wild-type strain xjL12 of *A. citrulli*, Rif^R^ | This lab |
| WT-EV | xjL12 containing blank pBBR1MCS-5 | This lab |
| Δ*envZ_A_*_c_ | In-frame deletion of *Aave_1583*, Rif^R^ | This study |
| Δ*envZ_A_*_c_-EV | IIn-frame deletion of *Aave_1583* containing blank pBBR1MCS-5, Rif^R^, Gm^R^ | This study |
| Δ*ompR_Ac_* | In-frame deletion of *Aave_1584*, Rif^R^ | This study |
| Δ*ompR_Ac_* -EV | In-frame deletion of *Aave_1584* containing blank pBBR1MCS-5, Rif^R^, Gm^R^ | This study |
| Δ*ompR_A_*_c_/*envZ_A_*_c_ | In-frame deletion of *Aave_1583* and *Aave_1584*, Rif^R^ | This study |
| Δ*ompR_A_*_c_/*envZ_A_*_c_-EV | In-frame double deletion of *Aave_1583* and *Aave_1584* containing blank pBBR1MCS-5, Rif^R^, Gm^R^ | This study |
| C*envZ_A_*_c_ | Complementary strain of Δ*envZ_Ac_*, containing pBBR-*envZ_A_*_c_, Rif^R^, Gm^R^ | This study |
| C*ompR_Ac_* | Complementary strain of Δ*ompR_Ac_*, containing pBBR-*ompR_Ac_*, Rif^R^, Gm^R^ | This study |
| C*envZ_Ac_*/*ompR_Ac_* | Complementary strain of Δ*envZ_Ac_/ompR_Ac_*, containing pBBR- *ompR_A_*_c_/*envZ_A_*_c_, Rif^R^, Gm^R^ | This study |
| Δ*envZ_A_*_c_^H266A^ | *envZ_Ac_* point mutation, the His266 substituted by Ala, containing pBBR-*envZ_A_*_c_^H266A^, Rif^R^, Gm^R^ | This study |
| Δ*ompR_Ac_*^D59A^ | *ompR_Ac_* point mutation, the Asp59 substituted by Ala, containing pBBR-*ompR_Ac_*^D59A^, Rif^R^, Gm^R^ | This study |
| *Escherichia coli* |  |  |
| DH5α | Φ80(*lacZ*)∆M15 ∆(*lac*ZYA-*arg*F)U169 *deo*R *rec*A1 *end*A1 *hsd*R17(rK^-^, mK^+^) *pho*A *sup*E44 λ^-^ *thi* -1*gyr*A96 *rel*A1, used for molecular cloning | Tsingke, Bejing, China |
| BL21(DE3) | *omp*T *hsd*S_B_(r_B_^-^ m_B_^-^) *gal* *dcm*(DE3), used for protein expression | Tsingke, Bejing, China |
| BW20676 | *Δpir pro hsdR*, *recA*, used for biparental mating | This lab |
| **Plasmids** |  |  |
| pK18*mobsacB* | Suicide vector used in in-frame deletion, Km^R^ | (Schäfer et al., 1994) |
| pBBR1MCS-5 | Broad-host-range vector used for genetic complementation, Gm^R^ | (Kovach et al., 1995) |
| pET30a | Protein expression vector, Km^R^ | This lab |
| pBBR-*nluc* | Luciferase reporter vector, containing a nano-luciferase (*nluc*) reporter gene, Gm^R^ | This lab |
| pK18- *envZ_A_*_c_ | The upstream and downstream of *envZ_A_*_c_ cloned into pK18*mobsacB* for deleting *envZ_A_*_c_, Km^R^ | This study |
| pK18- *ompR_A_*_c_ | The upstream and downstream of *ompR_A_*_c_ cloned into pK18*mobsacB* for deleting *ompR_A_*_c_, Km^R^ | This study |
| pBBR-*envZ_A_*_c_ | Recombinant vector containing the *envZ_A_*_c_ ORF for genetic complementation of *envZ_A_*_c_ mutant, Gm^R^ | This study |
| pBBR-*ompR_A_*_c_ | Recombinant vector containing the *ompR_A_*_c_ ORF for genetic complementation of *ompR_A_*_c_ mutant, Gm^R^ | This study |
| pBBR-*ompR_A_*_c_/*envZ_A_*_c_ | Recombinant vector containing the *ompR_A_*_c_/*envZ_A_*_c_ ORF for genetic complementation of *ompR_A_*_c_/*envZ_A_*_c_ mutant, Gm^R^ | This study |
| pBBR-*envZ_A_*_c_^H266A^ | Recombinant vector for constructing Δ*envZ_A_*_c_^H266A^, the His266 substituted by Ala, Gm^R^ | This study |
| pBBR-*ompR_A_*_c_^D59A^ | Recombinant vector for constructing Δ*ompR_A_*_c_^D59A^, the Asp59 substituted by Ala, Gm^R^ | This study |
| pET30-EnvZ_Ac_C | Protein expression vector for expressing cytoplasmic region of EnvZ_Ac_ tagged by 6$\times$His, Km^R^ | This study |
| pET30-EnvZ_Ac_C^H266A^ | Protein expression vector for expressing cytoplasmic region of EnvZAc tagged by 6$\times$His, with the His266 substituted by Ala, Km^R^ | This study |
| pET30-OmpR_Ac_ | Protein expression vector for expressing OmpR_Ac_ tagged by 6$\times$His, Km^R^ | This study |
| pET30-OmpR_Ac_^H266A^ | Protein expression vector for expressing OmpR_Ac_ tagged by 6$\times$His, with the Asp59 substituted by Ala, Km^R^ | This study |
| pBBR-P*ompR_Ac_*-*nluc* | Reporter vector for detecting activity of *Aave_1584* promoter, Gm^R^ | This study |
| pBBR-P*hrpG*-*nluc* | Reporter vector for detecting activity of *Aave_0445* promoter, Gm^R^ | This study |

^a^ Rif^R^, Km^R^, Gm^R^, Amp^R^, Cm^R^: rifamycin, kanamycin, gentamicin, ampicillin and chloramphenicol resistance, respectively; ORF: open reading frame

Kovach, M.E., Elzer, P.H., Hill, D.S., Robertson, G.T., Farris, M.A., Roop, R.M., et al. 1995. “Four New Derivatives of the Broad-Host-Range Cloning Vector pBBR1MCS, Carrying Different Antibiotic-Resistance Cassettes.” *Gene* **166**: 175–176.

Schäfer, A., Tauch, A., Jäger, W., Kalinowski, J., Thierbach, G., and Pühler, A. 1994. “Small Mobilizable Multi-Purpose Cloning Vectors Derived from the *Escherichia coli* Plasmids pK18 and pK19: Selection of Defined Deletions in the Chromosome of *Corynebacterium glutamicum*.” *Gene* **145**: 69–73.
