# Supplementary material for: Osmotic and pH Stress‐Responsive Two‐Component System, OmpR/EnvZ, Modulates Type III Secretion, Biofilm Formation, Swimming Motility and Virulence in Acidovorax citrulli xjL12
Source: Mol Plant Pathol. 2025 Jun 16;26(6):e70107. doi: 10.1111/mpp.70107 (PMC12170943; doi:10.1111/mpp.70107)
Supplement: Supplementary file 12 — Table S2. [file MPP-26-e70107-s011.docx]

**Table S2** Primers for PCR and RT-PCR in this study

| **Primers** | **Sequence (5′ to 3′) ^a^** | **Description ^b^** |
| --- | --- | --- |
| **PCR primers** |  |  |
| *1583*-F1 | gagctcggtacccggggatccCCCTTCAATCCCCGCGAA | Amplifies upstream and downstream fragments of *envZ_Ac_* to construct pK18- *envZ_A_*_c_ |
| *1583*-R1 | ccttcgcGGCGCTCATGTCAGGTCAGG |  |
| *1583*-F2 | tgacatgagcgccGCGAAGGCCTGAACAGCA |  |
| *1583*-R2 | acgacggccagtgccaagcttACTTTCTTCTGACAAGACACAC |  |
| *1584*-F1 | gagctcggtacccggggatccCTCCATCTGTTCGGGCTGC | Amplifies upstream and downstream fragments of *ompR_Ac_* to construct pK18- *ompR_A_*_c_ |
| *1584*-R1 | gccgtggatcaCATGAGTTTCCTGGGTCCTATTTG |  |
| *1584*-F2 | aaactcatgTGATCCACGGCCGACAAC |  |
| *1584*-R2 | acgacggccagtgccaagcttCTCGGTGAGGCGCACGCC |  |
| C*1583*-F | gtcgacggtatcgataagcttATGAGCGCCTCCCACGAC | Amplifies *Aave_1583* ORF to construct genetic complementary vector of Δ*envZ_Ac_*. |
| C*1583*-R | caggaattcgatatcaagcttTCAGGCCTTCGCCGGCTTGAC |  |
| C*1584*-F | agggaacaaaagctgggtaccGCGCACCTCCATCTGTTCG | Amplifies *Aave_1584* ORF to construct genetic complementary vector of Δ*ompR_Ac_*. |
| C*1584*-R | caggaattcgatatcaagcttTCGCTCGCTCCATCCGGCAC |  |
| C*1584*/*1583*-F | agggaacaaaagctgggtaccGCGCACCTCCATCTGTTCG | Amplifies *Aave_1584/Aave_1583* operon to construct genetic complementary vector of Δ*ompR_A_*_c_/*envZ_A_*_c_. |
| C*1584/1583*-R | caggaattcgatatcaagcttTCAGGCCTTCGCCGGCTTGAC |  |
| *1583*_H266A-F | CATCTCC**gc**CGACCTGCGCACGCCGCTGGCGCGCCT | Used for constructing point-mutated vectors of pBBR-*envZ_Ac_^H266A^* and pET30a-EnvZ_Ac_^H266A^ |
| *1583*_H266A-R | GCAGGTCG**gc**GGAGATGCCGGCCAGCATCACC |  |
| *1584*_D59A-F | CGTTCTGG**c**TCTCATGATGCCCGGCGAGGACG | Used for constructing point-mutated vectors of pBBR-*OmpR_Ac_*^D59A^ and pET30a-OmpR_Ac_^D59A^ |
| *1584*_D59A-R | TCATGAGA**g**CCAGAACGATCAGTTCGACCGTC |  |
| pET30- 1583C-F | taagaaggagatatacatatgGCGCGGCTCATCAACCGG | Amplifies C-terminal *envZ_Ac_* to express cytoplasmic region of EnvZ_Ac_ |
| pET30- 1583C-R | ctcgagtgcggccgcaagcttGGCCTTCGCCGGCTTGAC |  |
| pET30- 1584-F | taagaaggagatatacatatgGCCTCTACAACCAACCGTACCG | Amplifies *ompR_Ac_* to express OmpR_Ac_ |
| pET30- 1584-R | ctcgagtgcggccgcaagcttGCTCGCTCCATCCGGCAC |  |
| P*1584* -*nluc*-F | cgctctagaactagtggatccGCGCACCTCCATCTGTTCG | Amplifies the promoter of *ompR_Ac_* to construct report vector pBBR-P*ompR_Ac_ nluc* |
| P*1584*-*nluc*-R | ttcgagtgtgaagaccatatgGGCCATGAGTTTCCTGGGTC |  |
| P*0445*-*nluc*-F | cgctctagaactagtggatccTTCGATCGTCGCGGCCCC | Amplifies the promoter of *hrpG* to construct report vector pBBR-P*hrpG*-*nluc* |
| P*0445*-*nluc*-R | ttcgagtgtgaagaccatatgGACTCGCATGATTTCCCCATAC |  |
| P*1584*-F | CAGGGGGGCGAGGAATTTCAT | Amplifies the P*ompR_Ac_* probe for EMSA |
| P*1584*-R | CGGTTGGTTGTAGAGGCCAT |  |
| P*0445*-F | GCATGATTTCCCCATACGCAAACG | Amplifies the P*hrpG* probe for EMSA |
| P*0445*-R | GAGTCATATAGGAAAGTTATTGGTATCGG |  |
| Tn5-F | CTATCGATTGTATGGGAAGCC | Transposon-specific primers for identifying the Tn5-insertion site by sequencing |
| Tn5-R | CGAGGCAGTTCCATAGGATGG |  |
| F1 | GCACCAGCAGGTCCTGCTG | Used for determining the co-transcription of *ompR_Ac_* and *envZ_Ac_* |
| R1 | CCGTCTTCCGCCACCATGAC |  |
| F2 | GCGAAAAACTGGCCCTGCTG |  |
| R2 | GAATTCGAGCGCGCGCAGC |  |
| F3 | GGCAGCATCCTGGCCTGGC |  |
| R3 | GCGAAATCGCCGTCGCGCA |  |
| **RT-qPCR primers** |  |  |
| 16S-F | CCTACGGGAGGCAGCAG | Used for internal control in RT-qPCR |
| 16S-R | ATTACCGCGGCTGCTGG |  |
| *ompR_Ac_*-F | GCTTCGAAGTCATGGTGGC | Used for RT-qPCR to detect the expression level of corresponding genes |
| *ompR_Ac_*-R | ATGATGATCGGGGTGCGG |  |
| *hrpG*-F | ATCACGGTGGATGGCTGC |  |
| *hrpG*-R | GTGCGCTTGTTGGATTCGAC |  |
| *hrpX*-F | CTCTCTCCTCCCAACGCATC |  |
| *hrpX*-R | CTGTCCTATCAGCAGCAGGG |  |
| *hrcC*-F | TCGGCGCTGTATTTCTACCC |  |
| *hrcC*-R | GAGCTCACCAGTTCCACGTG |  |
| *hrcT*-F | GGTTGCTCGAGTCCACCAG |  |
| *hrcT*-R | GGGTTGGTCTGCTGGATGTT |  |
| *hpaB*-F | GGCTTCGAGGTCCAGATAGC |  |
| *hpaB*-R | ACGATGAGGATGATGCCGC |  |
| *hrpE*-F | GAATTCGAAACCGCCACCAC |  |
| *hrpE*-R | CTGATCGACCAGGGCTGC |  |
| *ompR_Ac_*-F | GCTTCGAAGTCATGGTGGC |  |
| *ompR_Ac_*-R | ATGATGATCGGGGTGCGG |  |
| *fliA*-F | TCGGCATGATCGGGCTCAAC |  |
| *fliA*-R | TTCTGGCTCTTGCGCGAACT |  |
| *fliC*-F | CCGCAACGTCTCGGAACTGA |  |
| *fliC*-R | CTGCGAGGACACGTCGTTGA |  |
| *fliD*-F | GCAAGATCAACGGCTCCAAG |  |
| *fliD*-R | GCATATTCAGTCGAGCCGGA |  |
| *flgK*-F | ACCGACATGCTCAACTCGTT |  |
| *flgK*-R | GAGCTGTTCCTTCACGGTGTA |  |
| *fliF*-F | TGTCCTACGTGCAGCAGATC |  |
| *fliF*-R | GAGCTTTCCACCACCTGCT |  |
| *motA*-F | ATCTTCGGGGTCTTCATCGC |  |
| *motA*-R | TAACGCGCCTTGGTGTACTT |  |

^a^ Nucleotides with lowercase represent overlap sequences for in-fusion clone. Nucleotides underlined represent restriction enzyme sites. Nucleotides in bold represent point mutant sites

^b^ RT-qPCR, real-time quantitative PCR; EMSA, electrophoretic mobility shift assay. ORF, open reading frame
